# Combinatorial mutagenesis of N-terminal sequences reveals unexpected and expanded stability determinants of the *Escherichia coli* N-degron pathway

**DOI:** 10.1101/2025.05.22.655665

**Published:** 2025-05-22

**Authors:** Sabyasachi Sen, Nastassja Corrado, Alexander Tiso, Khai Khee Kho, Aditya M. Kunjapur

**Affiliations:** Department of Chemical & Biomolecular Engineering, University of Delaware, Newark, DE 19716

## Abstract

Although it is known that penultimate N-terminal residues can influence protein stability by the N-degron pathway, there has not been a comprehensive effort to document nor predict these effects for natural and synthetic sequences in prokaryotes. Here, we present the deepest sequence coverage screen of the bacterial N-degron pathway using combinatorial site-saturation mutagenesis, fluorescence-activated cell sorting (FACS), and next-generation sequencing (NGS). Our results reveal new categories of exceptions and nuance to the N-degron pathway observed in bulk and context-specific analyses. For example, we find cases where penultimate residues can reinforce or shift the stability expected based only on the identity of position 1 (P1). We observe lowered stability for P2-P5 motifs rich in bulky residues and Gln as well as heightened stability for P2-P5 motifs rich in negatively charged residues, Gly, and Pro, particularly when these residues are at P2. We find that P1 Cys can be a component of an N-degron in a sequence-specific manner. Furthermore, we employ machine learning to predict protein stability, identifying several motifs with unexpected fates. Our work expands the sequence determinants of the bacterial N-degron pathway with unprecedented granularity, providing a set of sequence guidelines for proteomic investigation and protein engineering.

## Introduction

Protein degradation plays a key role in supporting natural and engineered functions of bacteria. Bacterial protein degradation pertains to human health in myriad ways, such as in the development of tools such as BacPROTACs^1^ that can guide antibiotic target discovery through inducible degradation of essential proteins. Furthermore, bacterial proteins directly involved in protein degradation, such as the Clp protease system in the pathogen *Mycobacterium tuberculosis*^2,3^, are attractive antibiotic targets. In applied biotechnology efforts, protein degradation tags are frequently used to attenuate hysteresis in genetic circuits that are expressed in industrial workhorse strains such as *Escherichia coli*^4–6^. Additionally, bacterial proteins that recognize and recruit N-terminal degrons (N-recognins) have also been repurposed for next-generation protein sequencing methods^7–10^.

**Figure 1.**
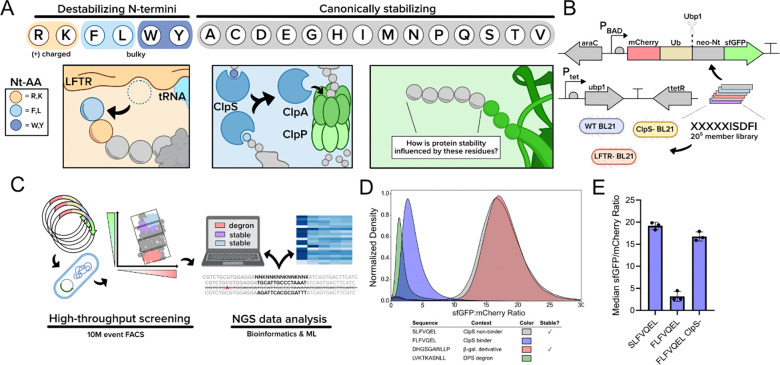
**A.** An overview of the *Escherichia coli* Leu/N-degron pathway. The top row shows the expected fate for proteins with the listed amino acids at the N-terminus, where the orange and blue shaded residues are canonically destabilizing. The left-most box illustrates the leucyl/phenylalanyl transferase LFTR, appending an F or L onto an N-terminal R or K. The middle box illustrates the ClpS adaptor protein that binds N-terminal F, L, W, and Y and recruits the protein to the ClpAP protease for degradation. The right-most box shows the dual reporter after the action of a ubiquitin (Ub) cleavase, revealing a neo-N-terminus (neo-Nt) on an sfGFP reporter. **B.** Genetic constructs for the protein stability assay. Wild-type or knockout *E. coli* strains were transformed to harbor two plasmids. The first plasmid harbors a synthetic gene fusion that encodes the dual fluorescent reporter under control of an arabinose-inducible promoter. The second plasmid harbors a gene encoding ubiquitin cleavase from *Saccharomyces cerevisiae* under control of a tetracycline-inducible promoter. Constructs were tested in wild-type BL21 as well as BL21 derivatives deficient in *clpS* and *aat* (LFTR). **C.** The HTS protein stability workflow used in this study. It includes 10^6^ sequence DNA library generation, high-efficiency transformation, 10^7^ event cell sorting, next-generation sequencing, and data analysis that includes machine learning, quality control, stability evaluation, and motif enrichment. **D.** The protein stability assay differentiates literature controls. sfGFP:mCherry ratio density histograms for over 100,000 events collected for cells expressing Ubp1 as well as literature control N-terminal sequences embedded in a dual fluorescent reporter construct. Stable sequences were SLFVQEL and DHGSGAWLLP and unstable sequences were FLFVQEL and LVKTKASNLL. Dual reporter ratiometric comparison of literature controls using flow cytometry. **E.** Known ClpS-binding degron FLFVQEL returns stabilizing sfGFP:mCherry ratio values in the absence of ClpS. Data shown is median fluorescence intensity ± standard deviation for at least 100,000 events captured across clonal biological triplicates using flow cytometry. Figure generated using Biorender content.

Protein degradation by the N-degron pathway, which is conserved across both prokaryotes and eukaryotes^11^, can modulate protein half-life by orders of magnitude in bacteria and is therefore of significant relevance to fundamental and applied researchers. In *E. coli,* bulky (Leu, Phe, Trp, Tyr) or positively charged (Arg, Lys) amino acids at the N-terminus serve as the primary signal for N-degradation based on recognition by one or both of two N-recognins^12,13^. The adaptor protein ClpS, a prokaryotic homolog of the eukaryotic N-recognin Ubr1^14^, recognizes and binds to N-degron-tagged proteins that carry bulky amino acids at the N-terminus and deposits them at the ClpAP protease, accelerating substrate turnover ^15–18^. A second N-recognin, LFTR (leucyl-phenylalanyl transferase), recognizes positively charged amino acids at the N-terminus and will append one or more Leu/Phe onto the N-terminus to generate a new substrate for ClpS^19–21^. However, the identity of the N-terminal residue is not a definitive determinant of protein fate. Recent work has shown that residues downstream of the N-terminus through at least the first 5 positions of a protein can impact stability through the pathway, including situations where the fate of a protein appears to defy its N-terminal residue^18,20,22–27^. These important yet relatively limited observations strongly motivate a comprehensive analysis of N-degron sequence stability determinants in bacteria (Figure 1a). Additionally, synthetic and chemical biologists have increasingly designed alternative protein N-termini for non-degradative purposes, such as for bio-orthogonal conjugation reactions specific to certain N-terminal amino acids^28–31^, live cell cleavage of N-terminal tags or signal peptides, or protein ligation or excision via inteins, sortases, or subtiligases^32–36^. For these applications and more, it is valuable to ensure that any generated neo-N-termini do not form an N-degron.

To date, the characterization of the bacterial N-degron pathway has been performed using low-throughput protein half-life assays, *in vitro* pathway reconstitution, and affinity purification to screen dozens to hundreds of putative N-degrons at a time ^12,19,20,24,26,37,38^. While these methods have pioneered our understanding of N-degrons, they have their limitations. Pulldown assays are limited to sequences found and enriched within the bacterial proteome. Low-throughput reporter screens require extrapolation from smaller data sets, and *in vitro* experimentation can sometimes present artifacts or other deviations from *in vivo* pathway behavior. To address these limitations, recent advances within the protein degradation field, including exemplary work by Timms et al.^39^, have leveraged high-throughput screening and next-generation of screening N-terminal libraries derived from the human proteome to analyze roughly 10^4^ sequences within human cells. Similar approaches have been taken to study protein degradation^40–42^, but no studies to date have screened large DNA libraries within bacteria to characterize the N-degron pathway.

To address this gap, we evaluated the role of the N-terminal 5 amino acids (P1-P5) using degenerate oligonucleotides for combinatorial mutagenesis, reporter protein expression in wild-type and N-recognin-deficient bacterial hosts, fluorescence-activated cell sorting (FACS), and next-generation sequencing (NGS). From over 2 million unique sequences screened and collected, we have mapped N-degron pathway with increased granularity, as visualized through position-by-position substrate specificity maps and motif analysis. We additionally share a series of sequence-based stability determinants. Specifically, at P2 Gln (destabilizing), Pro (stabilizing), and Gly (stabilizing) show the largest single residue stability shifts. Furthermore, additive stability effects were observed for motifs rich in bulky residues (destabilizing), negatively charged residues (stabilizing), and flexible Gly/Ser residues (stabilizing). In an analysis of low stability sequences without the canonically destabilizing P1 FLYWRK, we found motifs rich in P1 Cys and bulky and negatively charged residues in P2-P5. We then trained a machine learning model, N-FIVE, on our dataset and harnessed it to predict sequences that break canonicalstability rules. We conclude by discussing the implications of this work on pathway specificity, N-degron design, and future applications of our platform.

## Results

### Overview and development of an *in vivo* N-degron pathway fluorescent reporter

To monitor *in vivo* protein degradation, we adapted the ubiquitin fusion technique^43^ into a dual fluorescent reporter, creating an mCherry-ubiquitin-degron-sfGFP-His6x genetic fusion which we cloned into a plasmid (p15a ori, CmR, P_araBAD_). Ubiquitin (Ub), which is generally considered inert within *E. coli*, is scarlessly cleaved at its C-terminus upon expression of Ubp1, a Ub protease from *Saccharomyces cerevisiae*, using a second plasmid (ColE1 ori, KanR, P_tet_). This cleavage reveals a neo-N-terminus (neo-Nt) that is attached to sfGFP while simultaneously producing a stable fluorescent mCherry-ubiquitin motif. The subsequent degradation of a destabilizing motif attached to sfGFP should result in lower sfGFP:mCherry ratios for pathway substrates in comparison to stabilizing motifs that are attached to sfGFP (Figure 1b). This dual fluorescent reporter assay avoids various confounding effects, including variable protein expression due to 5’ ORF mRNA sequence and the amino acid sequence near the translational start site^44–52^. We selected *E. coli* BL21 as the expression host due to its deficiency of the Lon and OmpT proteases, helping to minimize proteolytic crosstalk and to isolate the effect of the ClpSAP system. To further isolate the impact of each key N-recognin and control for possible changes in reporter expression, we generated two separate variants of BL21 where either ClpS or LFTR were inactivated through the insertion of in-frame stop codons using multiplex automatable genome engineering^53,54^. This configuration paired with mutagenesis of neo-Nt regions serves as the basis for our high-throughput screening of the pathway (Figure 1c).

To verify assay functionality, we screened neo-Nt sequences previously reported to be strong and weak ClpS/N-degron pathway substrates. For positive controls, we selected reported ClpS ligand FLFVQEL^26,55^ and LVKTKASNLL, the latter derived from ClpSAP substrate Dps^20^. For negative controls, we selected SLFVQEL, a known ClpS non-interacting sequence, and DHGSGAWLLP^12^, a reported stable motif derived from the first 10 amino acids of β-galactosidase. Using flow cytometry, we observed distinct unimodal distributions of sfGFP/mCherry ratio across all tested degrons and selected median sfGFP/mCherry ratio as a comparative metric. Ratiometric comparisons revealed an average 6.0 and 5.9-fold dynamic range between SLFVQEL/FLFVQEL and DHGSGAWLLP/LVKTKASNLL pairs, respectively (Figure 1d). We additionally screened FLFVQEL in the ClpS^−^ host and observed a 5.2-fold ratio increase over the wild-type strain, further evidencing that observed ratiometric changes from our assay matched expectations for N-degron pathway mediated turnover (Figure 1e).

### Deep sequence profiling reveals penultimate residue stability determinants

**Figure 2:**
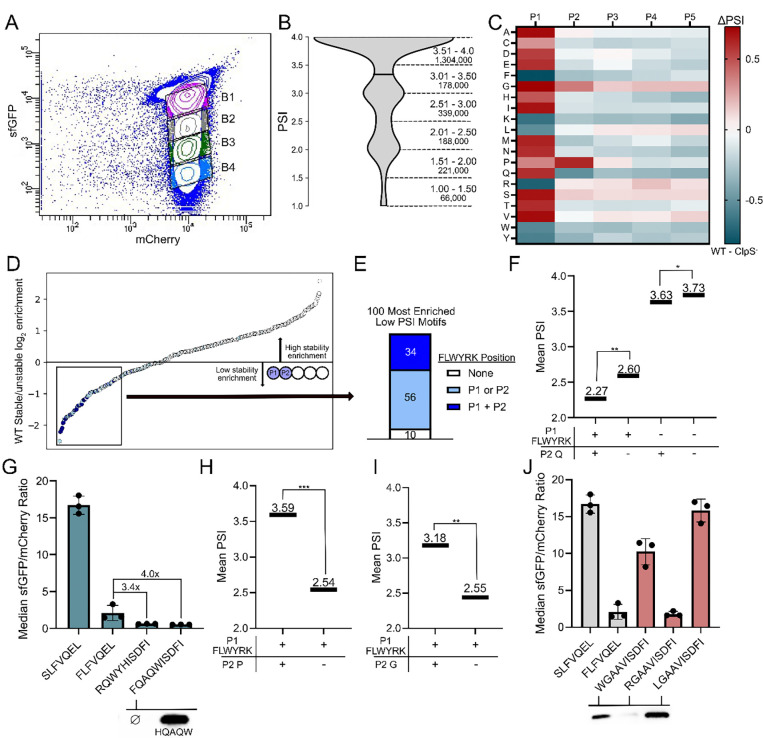
**A.** FACS binning of the XXXXXISDFI library within wild-type BL21 **B.** Violin plot showing the PSI distribution of 2.30M collected sequences from the wild-type BL21 dataset. The number of sequences rounded to the nearest thousand can be found below the corresponding PSI sub-range. **C.** Mean change in PSI heatmap between wild-type BL21 and ClpS− BL21. **D.** Log_2_ enrichment ratio plot of the 400 possible stable (PSI >3)/unstable (PSI<2) P1-P2 dipeptide motifs. Sequences containing LFWYRK in either P1 or P2 are highlighted in light blue and sequences containing LFWYRK in both P1 and P2 are highlighted in dark blue. **E.** LFWYRK composition of the 100 lowest PSI P1/P2 motifs. **F.** Mean PSIs for wild-type sequence subsets, revealing an amplified PSI lowering effect by P2 Gln when paired with a canonically unstable N-terminus. The number of sequences per condition are (++): 29,982, (+−): 754, 580, (−+): 52,021, (−−): 1,459,940; (++/+−) ES: 0.26. **G.** Clonal P2 Gln isolates show decreased stability measurements relative to a literature control. Flow cytometry data for various P2 glutamine motifs (top). Western blots of hardcoded sequences (bottom) No band was visible for the FQAQWISDFI sequence. **H.** Mean PSIs for wild-type sequence subsets, revealing an amplified PSI raising effect by P2 Pro when paired with a canonically unstable N-terminus. The number of sequences per condition are (++): 33,674, (+−): 750, 888; (++/+−) ES: 0.71. **I.** Mean PSIs for wild-type sequence subsets, revealing an amplified PSI lowering effect by P2 Gly when paired with a canonically unstable N-terminus. The number of sequences per condition are (++): 41,975, (+−): 742,587; (++/+−) ES: 0.47. **J.** Clonal P2 Gly isolates show stabilizing effects for two non-LFTR modified N-termini. Flow cytometry data for various P2 glycine motifs (top). Western blots of hardcoded sequences (bottom). Comparisons are abbreviated as (P1 FLYWRK presence, P2 presence). p-values evaluated using a Mann-Whitney U test. Effect size magnitude (ES) ranges from 0 to 1, as evaluated using Cliff’s Delta. * = p <0.05, ES 0–0.25; ** = p <0.05, ES 0.25–0.50, *** = p <0.05, ES 0.50–0.75, **** = p <0.05, ES 0.75–1.0

We next validated our N-degron screening platform on a 60-member library and profiled the P1-P3 stability of eukaryotic-derived N-degrons containing Nt-Arg-Cys, which led us to identify a strongly stabilizing influence of acidic residues at P3, which we verified by Western blotting^56^. After this validation on a small library, we sought to expand library size to the million-scale to determine the stability contributions for all combinations of the first five amino acids through the N-degron pathway. As the starting point for mutagenesis, we selected an N-degron derived from the eukaryotic protein RAP2.2 (CGGAIISDFI)^57^ due to its consistent behavior and high dynamic range between stable and unstable arginylated variants. Using 5 consecutive NNK codons located at the neo-N-terminus of the reporter (XXXXXISDFI, theoretical size: 3.2E6), we transformed the library into WT and knockout strains. We then induced expression of both reporter and Ubp1 and subsequently collected 10M gated events using fluorescence activated cell sorting (FACS). We sorted mCherry-expressing cells into four bins that covered over three orders of GFP fluorescence magnitude (Figure 2a). Upon analyzing 100–150M reads of an amplicon containing the mutagenized region from the WT, LFTR^−^, and ClpS^−^ sorts, we obtained 2.29M, 2.34M, and 2.19M unique sequences per host, respectively. To evaluate the stability of an analyzed sequence, we then calculated the Protein Stability Index (PSI), a weighted average recently pioneered in high-throughput protein degradation experimentation^39,58–60^. In line with expectations that most P1-P5 sequences are stable, PSI distributions were skewed towards high stability values, with over 82% of wild-type sequences being in the top half of the PSI range (Figure 2b).

To study the sequence-specific substrate preferences of each N-recognin, we next generated average PSI heatmaps for all amino acid and position combinations for the datasets resulting from expression in WT (Supplemental Figure 1), LFTR^−^(Supplemental Figure 2), and ClpS^−^ hosts (Supplemental Figure 3). We observed high consistency between datasets; in a comparison of the wild-type dataset and LFTR^−^ dataset, a linear correlation of PSI achieved an r-squared of 1.0 for the 17 amino acids that are expected to behave similarly when at P1 (Supplemental Figure 4). As expected, Arg and Lys at P1 exhibited differential stability between LFTR^−^ and WT hosts. In analyses of P1, Pro was excluded due to confounding effects where it is not uniformly cleaved by Ulp1. We also examined how a protease capable of modifying some neo-Nt that contain Met at P1, the native *E. coli* methionine aminopeptidase (MetAP), might affect assay results when the residue at P2 is expected to be destabilizing if at P1. We compared the PSI for motifs carrying Nt-Met (MXYYY) to those same motifs when they appear in the first four positions (XYYYZ) within the wild-type dataset. We observed that residues that are canonically destabilizing at P1 (L,F,W,Y,R,K) had increased mean PSI at the X-position in MXYYY motifs when compared to XYYYZ motifs (Supplemental Figure 5). In line with the published specificity of MetAP^61,62^, it did not appear that Met-LFWYRK motifs produced low PSI N-degrons in bulk, though the aminopeptidase may be functional on other motifs.

To study how individual amino acids impact stability as a function of position in the P1-P5 region, we computed the average PSI for each AA-position combination and plotted the differences between WT and ClpS^−^ hosts in a heatmap (Figure 2c). In line with the reported role of ClpS as a sequence-specific degradation initiator, the ClpS^−^ data shows sequence-agnostic parity in the absence of the N-recognin. All cells in the corresponding P1-P5 heatmap lied within 0.17 PSI units of each other, demonstrably smaller than the greater than 1 PSI differences observed between canonically stable and unstable P1 residues in WT BL21 and LFTR^−^ BL21. Furthermore, P1 stability trends match literature expectations. Notably, the PSI order for destabilizing residues is roughly tiered as Phe = Arg < Leu < Trp = Lys = Tyr from lowest to highest average PSI. An analysis of the 100,000 lowest PSI sequences revealed an enrichment for five of these six residues (excluding Lys) at and near P1 (Supplemental Figure 6).

**Figure 3.**
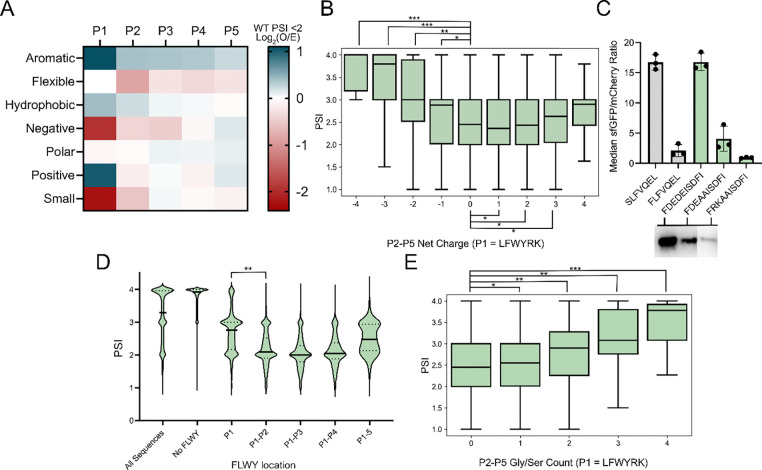
**A**. Low stability motif heatmap depicting log_2_ enrichment (observed/entire dataset) of grouped amino acids. Negative = DE, Positive = RK, Hydrophobic = AVILMFWY, Polar = RNDQEKHSTYC, Aromatic = FWYH, Small = GASC, Flexible = GSDN. **B.** Boxplot showing that sequences with P1 FLWYRK and negatively charged P2-P5 show increased bulk stability. (Net charge, n, ES relative to 0 net P2-P5 charge): (−4, 35, 0.62), (−3, 1,341, 0.61), (−2, 22,647, 0.39), (−1,155,936,0.15), (0,399,998, N/A), (+1, 171, 933, 0.04), (+2, 30,185, 0.01), (+3, 2,422, 0.07), (+4, 75, 0.24). All p-values in comparison to 0 net charge data show p <0.05. **c.** Negative charge can stabilize a canonically destabilizing P1 residue. (Top) Flow cytometry data for P1-P5 sequences with varying P2-P5 charge (FDEDE: −4, FDEAA: −2, FRKAA: +2). (Bottom) Western blots of corresponding sequences. **d.** Boxplot depicting that clustered FLWY at and near the N-terminus leads to decreased stability. x-axis lists exclusive locations for FLWY in the P1-P5 motif. P1(n=206,557) compared to P1+P2 (n=54,022) shows significance (p<0.05, ES = 0.45). **e.** Boxplot showing increased PSI means and distributions for sequences with P1 FLWYRK and increasing Gly/Ser residue counts in P2-P5. (Gly/Ser count, n, ES relative to 0 P2-P5 Gly/Ser): (0, 477,006, N/A), (1, 257,126, 0.07), (2, 46,829, 0.25), (3, 3,505, 0.50), (4,96, 72). All p-values in comparison to 0 P2-P5 Gly/Ser show p <0.05. p-values evaluated using a Mann-Whitney U test, effect size magnitude (ES) ranges from 0 to 1, as evaluated using Cliff’s delta. * = p <0.05, ES 0–0.25; ** = p <0.05, ES 0.25–0.50, *** = p <0.05, ES 0.50–0.75, **** = p <0.05, ES 0.75–1.0

Given the dominant role of P1 binding in stability determination, we next turned our focus towards the stability contribution of P2. As P2 is positioned on the periphery of the ClpS binding pocket in crystallized bound peptide ligands, docking studies have not clearly elucidated a role for this position (Supplemental Figure 7). First, we observed that select residues in P2 can generate highly destabilizing sequences in tandem with LFWYRK at P1. To this end, in an analysis of the 100 most enriched P1-P2 motifs at low PSI, 90/100 contained at least one FLWYRK residue, and 34/36 possible combinations containing two of FLWYRK residues were present, showing a clear overrepresentation of these six residues within destabilizing motifs relative to other sequence combinations clustered at P1-P2 (Figure 3d-3e).

We further observed that sequences containing Gln at or near P1 were amongst the most common sequences enriched at low PSI, which has not been previously reported to the best of our knowledge. More distinctly, Gln at position 2 (and to a lesser extent at P3–5) is amongst the most destabilizing P2 amino acid in both WT and LFTR^−^ BL21, providing a −0.51 PSI unit decrease at P2 in the WT dataset. This effect was amplified and significant when Gln was paired with P1 LFWYRK (p <1E-99, ES = 0.26), showing a 3.2x decrease in the difference in mean PSI compared to Gln paired with an alternative P1 residue (Figure 2f). The Gln trend was visible across WT and LFTR^−^ data and absent in the ClpS^−^ dataset, suggesting a ClpS-mediated preference (Supplemental Figures 1–3). Furthermore, amongst P1-P2 combinations that were enriched at low PSI but did not carry a canonically destabilizing P1 Gln was overwhelmingly present (Supplemental Figure 8). We sought to validate this finding using clonal examples. Upon pairing Gln with a destabilizing P1 residue we identified sequences that resulted in sfGFP:mCherry ratios that were 3.4 and 4.0 times lower below our previous degron control (RQWYHISDFI : FLFVQEL and FQAQWISDFI : FLFVQEL, respectively), and FQAQWISDFI showed no visible degron-sfGFP band when visualized via Western blot (Figure 2g).

Several amino acids showed stabilizing effects at P2. We observed this most distinctly from Gly and Pro, waning with distance from P1 with a maximal impact at position 2. Commonly enriched in stable, PSI >3 motifs (Supplemental Figure 9), these two residues had the highest PSI change in the (WT – ClpS^−^) heatmap. Notably, P2 Pro paired with P1 FLWYRK led to a significant increase 1.05-unit increase in PSI (p <1E-99, ES = 0.71) (Figure 2H). A similar, but smaller in magnitude 0.63-unit increase was observed for P2 Gly (p<1E-99, ES = 0.47) (Figure 2I). Upon testing motifs with P2 Gly, we found clonal isolates that can stabilize screened destabilizing N-terminal amino acids in Leu and Trp, while N-terminal Arg remained destabilizing, potentially due to Gly having an extended distance from the neo-P1 upon LFTR activity (Figure 2J).

### Similar residue side chains can cumulatively amplify or dampen N-degrons in an additive manner

We next investigated the combinatorial effect of multiple amino acids, hypothesizing that certain stability determinants could be a function of aggregate properties of amino acid sidechains, such as hydrophobicity or charge, or otherwise influenced by local sequence context. To select phenomena to target, we grouped amino acids based on R-group characteristics and evaluated their enrichment in low stability sequences within the WT dataset (Figure 3a). Unstable sequences showed an enrichment for aromatic, hydrophobic, and positively charged residues, and a deficiency in flexible, negatively charged, and small amino acids at various P2-P5 position, but most distinctly at P2. First, recognizing the differential enrichment in charged residues as well as the results presented in several papers that show that a penultimate charged residue can impact ClpSAP recognition and turnover^16,25,55^, we investigated whether there could be a relationship between the net charge of the N-terminal region and protein stability. We observed that lower PSI sequences are more commonly found in neutral to positively charged motifs, whereas negatively charged motifs, even with P1 FLYWRK, demonstrated large jumps in mean stability (Figure 3b). To further investigate, we tested clonal isolates carrying P1 Phe with motifs of various charge. We found FDEDE (−4) to be highly stabilizing and FDEAA (−2) to be lightly stabilized relative to a control degron or to a positively charged motif in FRKAA (+2) (Figure 3c). This charge-based phenomenon was absent in the ClpS^−^ dataset, suggesting that neutral to positive charges with P1 FLWYRK are critical to their recognition by ClpS (Supplemental Figure 10). Additionally, in the WT dataset both bulky (FLWY) and positively charged (RK) P1 residues showed increases in mean PSI for negatively charged P2-P5 motifs, though P1 RK sequences demonstrated a higher increase in mean PSI between neutral and negatively charged P2-P5 datasets (Supplemental Figure 11).

We next analyzed the impact of clustered FLWY residues in P2-P5 to inquire about the role of multiple bulky residues in this region. For sequences carrying multiple FLWY residues at and near the N-terminus, the average PSI was 0.56 units lower (p < 1E-99, ES = 0.45) for 2 residues compared to a singular P1 FLWY. Comparable distributions were observed for motifs carrying 2+ FLYW residues (Figure 3d). On average, sequences carrying a single positively charged residue at P1 exhibited a lower mean PSI than sequences with a bulky P1, though, notably, these sequences may have multiple bulky residues appended onto P1 Arg or P1 Lys via LFTR polyaddition^19,20^ (Supplemental Figure 12). We screened several clonal isolates with multiple bulky and positively charged residues at P1 + at least one of P2-P5 and found them all to be destabilizing (Supplemental Figure 13).

We additionally analyzed the additive impact of multiple smaller flexible amino acids, focusing on Gly and Ser, which frequently compose flexible N-terminal linkers. In line with the depletion of small amino acids in destabilizing motifs, we observed an increase in PSI with increasing Gly/Ser count downstream of a P1 FLWYRK residue (PSI increases for Gly/Ser counts 0–1: 0.09, 0–2: 0.32, 0–3: 0.64, 0–4: 0.99, all p<1E-33) (Figure 3e). As such, the presence of these flexible motifs may aid in the functionalization and stability of N-terminal motifs with canonically unstable residues.

### N-terminal cysteine can be destabilizing under specific sequence constraints

**Figure 4.**
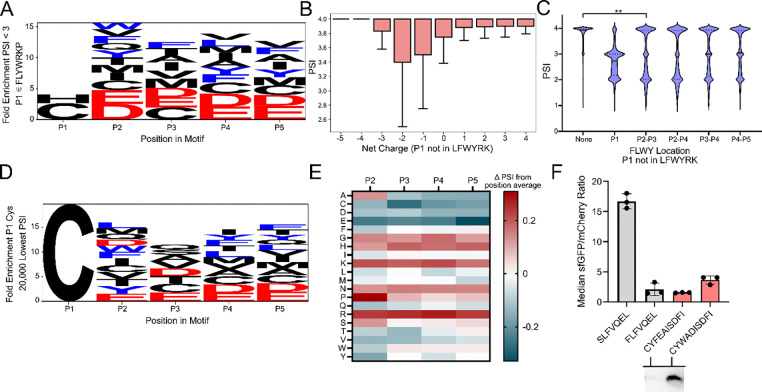
**A.** Low stability sequences with canonically stable N-termini are rich in negatively charged and bulky amino acids in P2-P5. Weblogo depicting fold enrichment amino acids in low PSI (PSI <3) motifs that do not contain FLWYRKP at P1. Bulky residues (FLWY) depicted in blue and negatively charged residues (DE) depicted in red. **B.** PSI boxplot for sequences with varying net charge and canonically stable P1 residues shows the opposite charge trend to low stability P1 FLWYRK sequences. P1 Pro is excluded. **C.** Multiple bulky residues lower mean PSI. Violin plot of wild-type BL21 data showing PSI distributions for sequences with bulky residues at various positions. A comparison between motifs with no bulky residues (n = 1,511,962) and motifs with bulky residues only in P2 and P3 (n=56,341) shows a significant difference between mean PSI values (p < 0.05, ES = 0.25) **D.** Weblogo depicting enriched amino acids for the 20,000 lowest PSI motifs with P1 Cys. Bulky residues (FLWY) depicted in blue and negatively charged residues (DE) depicted in red. **E.** Amino acid enrichment heatmap for motifs with P1 Cys. Change in PSI is calculated for each amino acid relative to the position average. **F.** Identification of an Nt-Cys degron. (Top) flow cytometry data, (bottom) Western blots of the degron-sfGFP product for candidate degrons with N-terminal cysteine. p-values evaluated using a Mann-Whitney U test, effect size magnitude (ES) ranges from 0 to 1, as evaluated using Cliff’s delta. * = p <0.05, ES 0–0.25; ** = p <0.05, ES 0.25–0.50, *** = p <0.05, ES 0.50–0.75, **** = p <0.05, ES 0.75–1.0

We next investigated whether there were generalizable sequence patterns for an N-degron with a canonically stable N-terminus (P1 not FLYWRK). To this end, we first analyzed amino acid enrichment in sequences with moderate to low stability (PSI < 3) that did not have LFWYRK at P1. Within these sequences, Cys and His were enriched at P1, and bulky residues (LFWY), acidic residues (DE), and other residues (MVC) were enriched at P2-P5 (Figure 4a). In line with the enrichment of acidic residues, we observed that canonically stable, low PSI motifs often maintained a neutral to negative charge (Figure 4b). Interestingly, low PSI sequences with noncanonically destabilizing P1 follow a charge preference that is opposite to that of the canonical six destabilizing P1 amino acids, with negatively charged motifs appearing more frequently at lower PSI. Furthermore, as previous studies have reported that bulky residues downstream of the N-terminus can be functionally destabilizing components of an N-degron^22^, we investigated sequence patterns with multiple bulky residues paired with a canonically stable P1 residue. We had observed that for sequences with bulky residues in P2/P3 that there was a bimodal distribution of sequences with high and low PSI (Figure 4c). In an analysis of low PSI sequences with bulky residues at P2 and P3, we observed that sequences with Gln, Cys, or His at P1 had the lowest average PSIs (Supplemental Figure 14). The largest decreases in PSI were observed for canonically stable P1 sequences with both bulky and negatively charged residues (Supplemental Figure 15).

**Figure 5.**
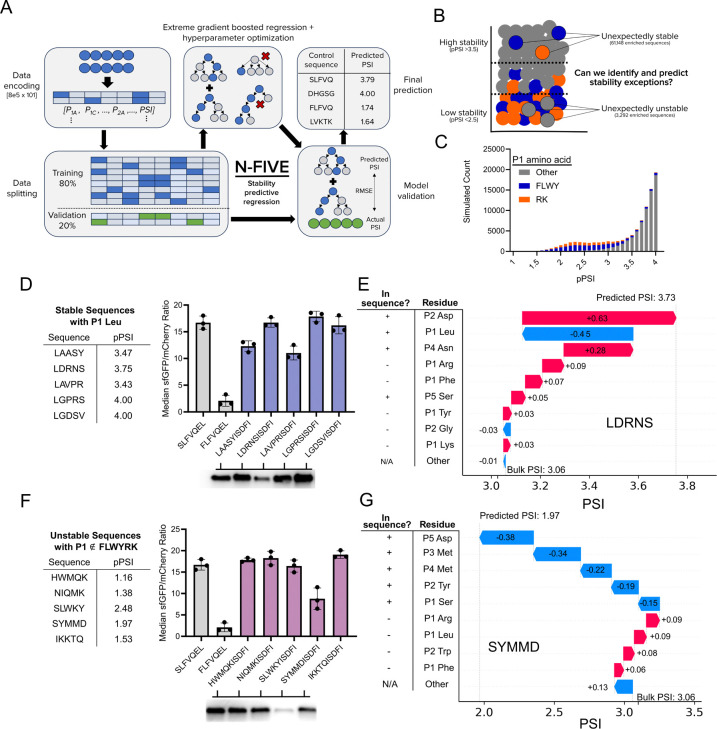
**A.** Architecture of the N-FIVE machine learning model. Enriched sequences were one-hot encoded into a matrix. 80% of sequences were used to train and optimize an extreme gradient boosted machine learning model. Model validation was performed on the remaining 20% of sequences. The first 5 amino acids of control sequences (Figure 1d) were predicted using N-FIVE and found to match the expected stability profile. **B.** High stability sequences (predicted PSI >3.5) are under-enriched in P1 LFWYRK and low stability sequences (predicted PSI <2.5) are over-enriched in P1 LFYWRK. Stability exception predictions would be a challenging task. **C.** PSI prediction of 100,000 randomly selected 5-amino acid sequences using N-FIVE. Sequences with a canonically destabilizing P1 are highlighted (bulky residues in blue, positively charged residues in orange). **D.** (Left) Predicted PSI values for five unexpectedly stable (pPSI >3.5) candidate sequences selected with a P1 Leu constraint. (Top right) Candidate sequence flow cytometry data, (right left) Western blots of the degron-sfGFP product. **E.** (right) SHAP values for individual matrix contributions to the predicted PSI value of 3.73 for the candidate sequence LDRNS. **F.** Predicted PSI values for five unexpectedly unstable (pPSI < 2.5) candidate sequences selected with a P1 ∉ FLWYRK constraint. (Top right) Candidate sequence flow cytometry data, (bottom right) Western blots of the degron-sfGFP product. **G.** (right) SHAP values for individual matrix contributions to the predicted PSI value of 1.97 for the candidate sequence SYMMD.

We next turned our attention to P1 Cys to understand what downstream residues could make this synthetically useful P1 residue an N-degron. Interestingly, P1 Cys had a lower mean PSI (3.42) compared to other stable P1 residues (PSI 3.80), suggesting that there was a subset of unstable sequences (Supplemental Figure 1). Upon visualizing the sequence composition of the 20,000 lowest PSI P1 Cys motifs, we observed an enrichment of bulky and negatively charged residues, whereas positively charged and flexible residues as well as P2 Pro were depleted in these motifs (Figure 4d). These enrichment trends aligned with residue-by-position PSI changes (Figure 4e). We generated clonal isolates of five sequences that carried candidate destabilizing motifs with P1 Cys that had bulky and negatively charged residues in P2-P5. Two of the five candidates presented sfGFP:mCherry ratios on par with the FLFVQEL degron, while the remaining three offered intermediate ratio values (Supplemental Figure 16). When we performed Western blotting analysis, at least one candidate sequence (CFYEAISDFI) was fully degraded, producing a non-visible band on par with the strongest identified degrons (Figure 5f).

We additionally investigated other commonly functionalized P1 residues in Gly and Ser. Both residues were notably stable at P1, though an analysis of P2-P5 residues for the 20,000 lowest PSI sequences for P1 Gly and P1 Ser revealed an enrichment in Gln, Glu, and Lys at P2-P5. (Supplemental Figure 17).

### Training and validation of N-FIVE, an N-terminal stability predictive model

To interpret the full sequence space, our next objective was to develop a stability-predictive machine learning model. During training, we observed increased statistical performance of our models upon applying a minimum read count threshold of 20 (RC>20). As such, we applied this filter to trim our WT dataset to approximately 798,000 sequences prior to final model training. To generate a base matrix, we one-hot encoded each enriched motif by amino acid and position. We then trained an extreme gradient boosted model using XGBoost. We supplied these encodings and the PSI from 80% of the WT dataset to generate the input for training. After hyperparameter tuning, our model obtained an RMSE of 0.34 and an r-squared of 0.82 when validated using the remaining 20% of data. Satisfied with the performance of our model, we dubbed our model N-FIVE and set out to test its predictive capabilities (Figure 4a-b). We first simulated the PSI of 100,000 random N-terminal sequences and observed the characteristic decrease in mean PSI for sequences carrying destabilizing N-termini (Figure 4c). Upon simulating the full 3.2M sequences using N-FIVE, we observed a high correlation compared to our actual dataset (Supplementary Figure 18). We generated SHAP plots to understand the underlying architecture, observing trends on par with known N-degron pathway rules such as the dominant role of P1 identity (Supplementary Figure 19).

To validate the predictive capabilities of N-FIVE, we sought to generate N-terminal motifs with unexpected stability profiles. To this end, we sought to identify five sequences with a canonically destabilizing P1 Leu that would have stable profiles. Excitingly, all five of the sequences (sequence LXXXXISDFI) suggested by N-FIVE displayed stable profiles (Figure 4d). We additionally investigated the challenging task to identify sequences that are unexpectedly unstable with P1 residues that are not one of FLWYRK. From our five-sequence screen, one motif, SYMMDISDFI, generated a moderately destabilizing profile (Figure 4f). SHAP values were calculated for candidate sequences LAASYISDFI (Figure 4e) and SYMMDISDFI (Figure 4g), revealing the cumulative effect of P2-P5 residues in overriding the P1 residue as a stability determinant.

## Discussion

This work presents the most comprehensive mapping of the *Escherichia coli* N-degron pathway to date. Having collected and analyzed over 2.2M screened sequences in the P1-P5 space within wild-type BL21, we both reinforce and provide insight into the pathway with increased granularity. Our findings corroborate the consensus understanding of the pathway within *E. coli*, namely that six amino acids (Leu, Phe, Tyr, Trp, Arg, Lys) when present at P1 by and large lead to protein degradation through the ClpSAP proteolytic cascade. We have expanded this to demonstrate how residues in the P2-P5 positions can augment or dampen P1 effects, in rare cases even fundamentally altering the expected level of protein stability. This is most evident for the destabilizing P2 Gln and the stabilizing P2 Pro and Gly. Notably, we observe that most destabilizing N-terminal motifs frequently have multiple bulky and positively charged residues in P1-P5. Exceptions to this rule frequently carry P1 Cys and His and have bulky or negatively charged residues in P2-P5. It is unclear how P2-P5 bulky amino acids may be causing instability as there is conflicting evidence about whether bulky residue downstream of P1 can be bound directly in the hydrophobic pocket of ClpS^22^. It is possible that this instability could be caused by strengthened interactions in the substrate channel or surface of the protein. Furthermore, in an analysis of N-degrons with canonically stable N-termini, we observed an enrichment in Cys, Gln, and His at P1.

Amongst the most destabilizing motifs that we observed were those that could be generated by LFTR. Multiple previous studies have identified that polyaddition of Leu and Phe, rather than addition of a singular Leu or Phe, is a possibility.^19,20^ The highly destabilizing nature of multiple Leu/Phe-Arg residues in tandem coincide with previous observations that the turnover of LR-substrates is greater than just L-substrates.^16^ To this end, others have suggested that the ClpS binding site has been optimized to accommodate LFTR-modified substrates.^63^ On balance, the generation of a highly destabilizing motif in front of Nt-Arg and Lys may routinely contribute to the generation of some of the most highly destabilizing degrons, demonstrating the rapid turnover of LFTR-substrates despite the requirement of an additional modification.

These findings bear relevance to the design of synthetic N-termini for applications in small molecule or protein ligation in live cells. For example, bioconjugation efforts that rely on a P1 Cys should avoid bulky and negatively charged residues near the N-terminus. Gly-Ser motifs are likely to provide a stabilizing effect that would offset any degradation-promoting elements. The overrepresentation of negatively charged residues and bulky residues in low PSI sequences with stable N-termini and small residues in high PSI sequences is worth considering when designing protein neo-N-termini with targeted stability levels in mind. For such designs of synthetic neo-N-termini, the stability-predictive N-FIVE model can be consulted, including for design with sequence constraints. Our confidence in the predictive power of N-FIVE is evidenced by its ability to solve a complex sequence-stability problem – in this instance successfully suggesting unexpectedly stable sequences. The identification of unexpectedly unstable sequences remains a challenge, in part due to the small number of candidate sequences to form the dataset. Further development of more complex ML models may improve stability prediction capabilities.

## Methods

### Strains, plasmid cloning, and transformations

*Escherichia coli* strains and plasmids used are listed in Table S1 and Table S2, respectively. *clpS* and *aat* inactivations were performed as described in the Genetic knockout of N-recognins section. Genes were purchased as G-Blocks or gene fragments from Integrated DNA Technologies (IDT) or Twist Bioscience and were optimized for E. coli K12 using the IDT Codon Optimization Tool. A version of scUbp1 without the N-terminal region was used for improved expression in *E. coli*^64^. Essential genetic sequences and primers can be found in Table S3 and Table S4, respectively. Cloning amplicons were generated using KOD XTREME Hot Start polymerase and corresponding buffering reagents. Amplicons were verified and purified by running through a 1% agarose gel for 200 V for 20 minutes followed by gel excision and extraction. Samples were assembled in homemade Gibson assembly aliquots run at 50 °C for 30 minutes. Assemblies were transformed into *E. coli* cloning strains DH5α or DH10β using either electroporation or chemical heat shock. Following a one-hour outgrowth after transformation, cells were plated on permissive antibiotic plates and were incubated at 37 °C overnight. The following afternoon, multiple colonies were picked and incubated overnight in separate culture tubes containing LB media (10 g/L tryptone, 5 g/L sodium chloride, 5 g/L yeast extract) and the permissive antibiotic. The next day, cells were stocked 1:1 with 30% glycerol at negative 80 °C for future usage. The remaining cells were miniprepped for sequencing. Plasmid sequences were verified through a mixture of Sanger sequencing through Eurofins, Genewiz, and Azenta and full plasmid sequencing through Plasmidsaurus. Plasmids were transformed into BL21 and BL21 derivatives using either electroporation or chemical heat shock. Two plasmid systems were transformed in series.

### Materials and chemicals

The following compounds were purchased from MilliporeSigma (Burlington, MA, USA): phosphate-buffered saline (PBS), kanamycin sulfate, glycerol, 25 nm membranes (VSWP02500) and KOD XTREME Hot Start polymerase (Millipore 71975–3). D-glucose was purchased from TCI America (Portland, OR, USA). Agarose, Laemmli SDS sample reducing buffer, and ethanol were purchased from Alfa Aesar (Ward Hill, MA, USA). Anhydrotetracycline (aTc) was purchased from Cayman Chemical (Ann Arbor, MI, USA Methanol, sodium chloride, LB Broth powder (Lennox), LB Agar powder (Lennox), Amersham ECL Prime chemiluminescent detection reagent, and Thermo Scientific^™^ Spectra^™^ Multicolor Broad Range Protein Ladder were purchased from Fisher Chemical (Hampton, NH, USA). Taq DNA ligase was purchased from GoldBio (St. Louis, MO, USA). Phusion DNA polymerase and T5 exonuclease were purchased from New England BioLabs (NEB) (Ipswich, MA, USA). SybrSafe DNA gel stain was purchased from Invitrogen (Waltham, MA, USA). E. cloni 10G Supreme electrocompetent cells were purchased from Biosearch Technologies. Miniprep kits were purchased from Zymo. 4–20% precast protein gels were purchased from Bio-Rad. Flow cyometry filter caps (Chemglass Life Sciences CLS4380009), arabinose, DpnI enzyme (FERFD1704) and Immobilon-E Western blot membranes were obtained from Fisher Scientific. HRP-conjugated anti-6*His antibody (Proteintech HRP-66005) was obtained from Proteintech (Rosemont, IL, USA).

### Genetic knockout of N-recognins

Multiplex automatable genome engineering (MAGE) was used to inactivate the endogenous *aat* (LFTR) and *clpS* genes^65^. MAGE oligonucleotides were designed using MODEST^66^ to insert three in-frame stop codons into the gene of interest. Freshly made electrocompetent cells were resuspended in 5 μM oligonucleotide and subsequently electroporated to enable cell permeation of the oligonucleotide. Cells were outgrown in LB media for an hour prior to plating on permissive plates. To verify the desired genetic knockouts, allele-specific colony PCR was performed using KAPA 2G Fast HotStart polymerase (Roche KK5801). Additional Sanger sequencing was performed to verify asPCR hits.

### Fluorescence analysis of degrons

Overnights made from biological triplicate colonies of BL21 and recognin-deficient BL21 derivatives carrying both the dual reporter and Ubp1 expressing plasmids were inoculated at a 1:100 ratio in fresh LB media containing 0.2% arabinose, 100 ng/mL aTc, 20 μg/mL chloramphenicol, and 15 μg/mL kanamycin. Cells were grown at 37 °C for 18 hours prior to analysis. Cells were then filtered using 35 μm filter caps into cytometry tubes containing fresh PBS at a roughly 1:250 ratio of cell culture: PBS. Cells were analyzed on a NovoCyte Flow Cytometer (Agilent Technologies). Prior to analysis, scatter gates were used to isolate singlet bacterial cells of the appropriate morphology. sfGFP fluorescence was analyzed using a 488 nm laser and a 530/30 nm bandpass filter and mCherry fluorescence was analyzed using a 488 nm laser and a 660/20 nm bandpass filter. For all analyses, at least 100,000 events that passed through the singlet gate were collected.

### Western blotting of hexahistidine-tagged reporter proteins

1 mL of overnighted culture containing expressed dual reporter and Ubp1 protease were lysed using glass beads for 15 minutes. The sample was transferred to a fresh microcentrifuge tube and centrifuged at 12,000 RPM for 10 minutes. Subsequently, the supernatant was collected. Sample concentration was evaluated using a Bradford assay on a SpectraMax i3x with a BSA calibration curve. Sample concentration was normalized to 0.1 mg/mL in water with 1x SDS PAGE loading dye and denatured at 95 °C for 10 minutes. To separate proteins by size, 10 uL of sample is loaded into a 4–20% Mini-PROTEAN TGX gel and run at 180 V for 35 minutes. Subsequently, the gel is transferred onto an Immobilon membrane through wet transfer overnight (25V for 15 hours on ice). The following day, the membrane is blocked for at least one hour with 5% milk in TBST buffer. An HRP-conjugated anti-His6x antibody is added to the solution at a 1:10,000 dilution and is allowed to incubate at room temperature for 1 hour. Following three washes in TBST, ECL Prime chemiluminescent reagent is added to the membrane. Following a 10 second incubation, chemiluminescent images at varying intensities are captured using an Azure c280.

### DNA library preparation

DNA library plasmids were designed to be assembled from two separate amplicons. Primers containing 1–5 consecutive NNK codons were ordered from IDT, using a 25A/25C/25G/25T (N) or 50G/50T (K) hand-mixed nucleotide split to maximize diversity in the mutagenized region. PCR amplicons were generated using KOD XTREME polymerase using roughly 200 ng of plasmid template per reaction with an annealing temperature of 59 °C and extension time of 2 minutes for inserts and 3 minutes for the backbone. To maximize sequence diversity and avoid overamplification, 5 separate PCR amplicons were generated at a low cycle count (20 cycles) and high volume (50 μL per reaction). Amplicons were then digested using DpnI to remove the template. The amplicons were then isolated on a 1% agarose gel and subsequently purified using gel extraction. Amplicons were pooled and assembled using an isothermal Gibson assembly method with a total of 300 ng of total DNA at a 1:1 molar ratio of insert: backbone. Assemblies were then dialyzed on a 25 nm membrane placed over deionized water for 60 minutes. Five separate assemblies were pooled and then electroporated into E. cloni 10G Supreme electrocompetent cells. Immediately following electroporation, cells were mixed with 500 μL of SOC media and were placed in a culture tube in a 250 RPM shaking incubator set to 37 °C. Following a 30-minute outgrowth, cells were plated at serial dilutions to verify transformation efficiency. For 20^5^ member libraries, a transformation efficiency of 10^8^ colonies/transformation was the minimum threshold for further experimentation. Following a second 30-minute outgrowth, 5 mL of SOC media was added to the tube and cultures were allowed to grow overnight.

The following morning, library stocks were made by mixing 500 μL of undiluted culture at a 1:1 ratio with 30% glycerol, which were then stored at −80 °C. The remaining 4–4.5 mL of culture was miniprepped using a Zymo ZR Plasmid Miniprep-Classic kit. BL21 and recognin-deficient BL21 derivatives carrying a Ubp1-expressing plasmid were made electrocompetent using standard molecular biology procedures, concentrating 10 mL cultures to 200 μL competent cell aliquots. Miniprepped libraries were electroporated into freshly made competent cells and followed parallel outgrowth, efficiency measurements, overnight growth, and freezer stocking to original library transformations. A transformation efficiency of 10^8^ was obtained for all conditions. Library diversity was confirmed prior to sorting using 2.5 Mbp next-generation sequencing through Azenta’s AmpliconEZ platform.

### Cell culturing and fluorescence activated cell sorting

Overnights of BL21 and recognin-deficient BL21 derivatives carrying both library plasmids and Ubp1-expressing plasmids were inoculated at a 1:100 ratio in fresh LB media containing 0.2% arabinose, 100 ng/mL aTc, 20 μg/mL chloramphenicol, and 15 μg/mL kanamycin. Cells were grown at 37 °C for 18 hours prior to analysis. Cells were then filtered using 35 μm filter caps into cytometry tubes containing fresh PBS at a roughly 1:250 ratio of cell culture: PBS. Cells were sorted on a BD FACS Aria Fusion using a 70 μm or 85 μm nozzle and a neutral density 1.0 filter. Prior to analysis, scatter gates were used to isolate singlet bacterial cells of the appropriate morphology. sfGFP fluorescence was analyzed using a 488 nm laser and a 530/30 nm bandpass filter and mCherry fluorescence was analyzed using a 561 nm laser and a 610/20 nm bandpass filter. Sorting conditions and sample concentrations were optimized to obtain event rates on the order of 1,000 – 5,000 events per second and a sort efficiency >95%. 10 million gated cells were sorted into four equally sized bins covering the sfGFP range, excluding a small gap at the bottom of the distribution that was observed to have a higher concentration of debris and GFP-inactivating mutants. Cells were recovered overnight in a mixture of 1:1 recovered cells: LB media containing 1% glucose. The subsequent day, cells were stocked and miniprepped using methods described in the DNA library preparation section.

### Next generation sequencing sample preparation

250 base pair (AVITI) and 405 base pair (Amplicon EZ) DNA amplicons for next-generation sequencing were designed to contain a 5’ and 3’ sequencing adapter, a 3 base pair barcode for demultiplexing, and a centralized mutagenized region to ensure coverage by both paired-end reads. The full sequence can be found in our corresponding methods publication^56^. Amplicons were generated using KAPA 2G polymerase, minimizing cycle counts (20 was default) to decrease over-enrichment of select sequences. The amplicons were then isolated on a 1% agarose gel and subsequently purified using gel extraction. Samples were then submitted to Azenta’s Amplicon EZ service (~100,000–300,000 2×250 paired end reads) or to the UD Sequencing & Genotyping Center (~100,000,000 – 150,000,000 2×150 paired end reads on an Element AVITI).

### Next generation sequencing analysis

NGS data was first converted from FASTQ.gz format to FASTA for ease of analysis. When necessary, reads were demultiplexed using the three base pair barcodes that corresponded to unique sorted bins. Reads were compared to the consensus sequence for the submitted amplicon. Any reads that varied from the consensus sequence (excluding the barcode and mutagenized region) or those that contained stop codons (TAG, TAA, TGA) in the mutagenized region were then excluded from subsequent analysis. The mutagenized region was identified from each read based on a 15 base pair consensus sequence located directly after the mutagenized region and translated into an amino acid sequence. Read counts for each 5 amino acid motifs across the four bins of any given strain. From this data, the protein stability index (PSI) was calculated using the formula $PSI=\sum_{i=1}^{4}R_{i}\;*\;i$, where $R_{i}$ is the fraction of reads present in bin i^58^. Each unique sequence, its read counts per bin, and PSI were stored in a Pandas database for subsequent analysis.

For general enrichment analysis, the counts of any given residue or motif were first measured. To calculate enrichment differences between two populations, the average occurrence of an amino acid or motif in the appropriate population was calculated and subsequently subtracted to obtain a difference. To obtain a ratio, groupings were normalized to the same sample size and subsequently divided for ratiometric comparison. Log_2_ analysis was performed to simplify data visualization.

Heatmaps, boxplots, and violin plots were visualized using the Seaborn and matplotlib Python packages^67,68^ as well as GraphPad Prism. Weblogos were generated using Logomaker^69^. All code was developed in Python. Code, sequence databases, and N-FIVE are accessible on GitHub: https://github.com/KunjapurLab/N-terminal-cluster-stability. Full NGS data will be made available prior to publication.

### Development of N-FIVE machine learning model

During model training, we observed that applying a 20 read minimum filter minimized RMSE and improved R^2^ for the model. Utilizing the sequence-PSI database, wild-type BL21 data were one-hot encoded into an 798,837 × 101 matrix. The first 100 columns represented a binary encoding of each of the 20 amino acids across 5 positions, while the final column corresponded to the PSI value. The dataset was randomly split, with 80% of the data being used for model training and 20% being used for model validation and evaluation. An extreme gradient boosted nonlinear regression model was then trained using the Python XGBoost^70^ package for 20 rounds. Hyperparameter optimization was performed using Optuna^71^ over 30 trials, aiming to minimize RMSE. The following parameters were set: ‘n_estimators’: 986, ‘max_depth’: 11, ‘learning_rate’: 0.085, ‘subsample’: 0.904, ‘colsample_bytree’: 0.810, providing an RMSE of 0.34. The final model was then saved as a .pkl file for future analysis. To evaluate the contribution of each matrix component, SHAP (Shapley Additive exPlanations) values were calculated^72^ for the whole model as well as for individual sequences.

### Statistical analysis

To quantify differences in PSI distributions between defined groups, we applied two non-parametric statistical methods: the Mann-Whitney U test and Cliff’s Delta for effect size calculations. The Mann-Whitney U test was selected due to the skewed nature of PSI distribution as well as its robustness to unequal sample sizes. We used a two-sided Mann–Whitney U test to assess whether the PSI distributions between two groups differed significantly. Cliff’s Delta was computed to quantify the magnitude and direction of the distributional shift between compared groups. Unlike common effect size measurements such as Cohen’s d, Cliff’s Delta makes no assumptions about the underlying distribution of PSI values and remains valid for skewed, bounded, or ordinal data. Here, Cliff’s Delta represents the probability that a randomly selected value from one group is greater than a randomly selected value from the other, minus the reverse probability to yield an effect size bounded between −1 and +1. For clonal isolate testing, fluorescence measurements from biological triplicates were taken for at least 100,000 gated cells using flow cytometry. In line with common practices in the field, median fluorescence ratios +/− standard deviation were reported.

## Supplementary Material

Supplement 1

## Code availability

NGS analysis code, sequence databases, and the N-FIVE model have been made available at GitHub (https://github.com/KunjapurLab/N-terminal-cluster-stability).
