# Supplementary material for: Combinatorial mutagenesis of N-terminal sequences reveals unexpected and expanded stability determinants of the Escherichia coli N-degron pathway
Source: bioRxiv. 2025 Jul 15:2025.05.22.655665. Originally published 2025 May 22. Preprint. [Version 2] doi: 10.1101/2025.05.22.655665 (PMC12258705; doi:10.1101/2025.05.22.655665)
Supplement: Supplement 1 [file NIHPP2025.05.22.655665v2-supplement-1.pdf]

bioRxiv preprint doi: https://doi.org/10.1101/2025.05.22.655665; this version posted July 15, 2025. The copyright holder for this preprint (which was not certified by peer review) is the author/funder, who has granted bioRxiv a license to display the preprint in perpetuity. It is made available under aCC-BY 4.0 International license.

Supplemental File 1. Supplemental Figures 1-6 and Supplemental Tables 1-2

bioRxiv preprint doi: https://doi.org/10.1101/2025.05.22.655665; this version posted July 15, 2025. The copyright holder for this preprint (which was not certified by peer review) is the author/funder, who has granted bioRxiv a license to display the preprint in perpetuity. It is made available under aCC-BY 4.0 International license.

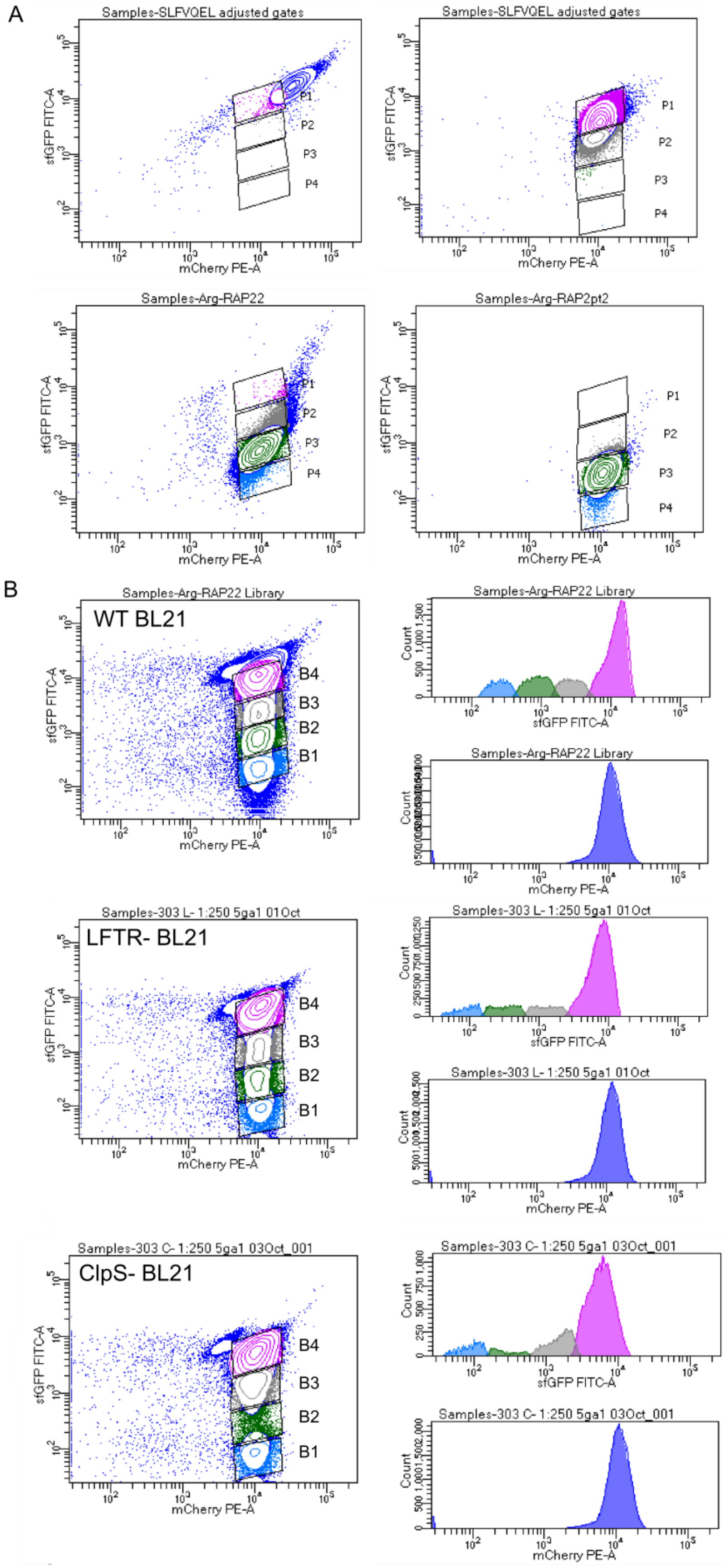

bioRxiv preprint doi: https://doi.org/10.1101/2025.05.22.655665; this version posted July 15, 2025. The copyright holder for this preprint (which was not certified by peer review) is the author/funder, who has granted bioRxiv a license to display the preprint in perpetuity. It is made available under aCC-BY 4.0 International license.

Supplemental Figure 1. A.) Differentiation of stable/unstable sort controls. SLFVQEL (stable, top) and RCGGAIISDFI (unstable, arginylated library template, bottom) sequences hardcoded into the dual fluorescent reporter are differentiated across two separate examples on a BD FACS Aria Fusion. Left samples were collected prior to the WT 10M event sort. B.) 10M event sort information. sfGFP (y-axis) vs. mCherry (x-axis) distributions for the sorted WT (top), LFTR$^-$ (middle), and ClpS$^-$ (bottom) libraries with corresponding bins (left). Histograms for sfGFP & mCherry fluorescence (right).

bioRxiv preprint doi: https://doi.org/10.1101/2025.05.22.655665; this version posted July 15, 2025. The copyright holder for this preprint (which was not certified by peer review) is the author/funder, who has granted bioRxiv a license to display the preprint in perpetuity. It is made available under aCC-BY 4.0 International license.

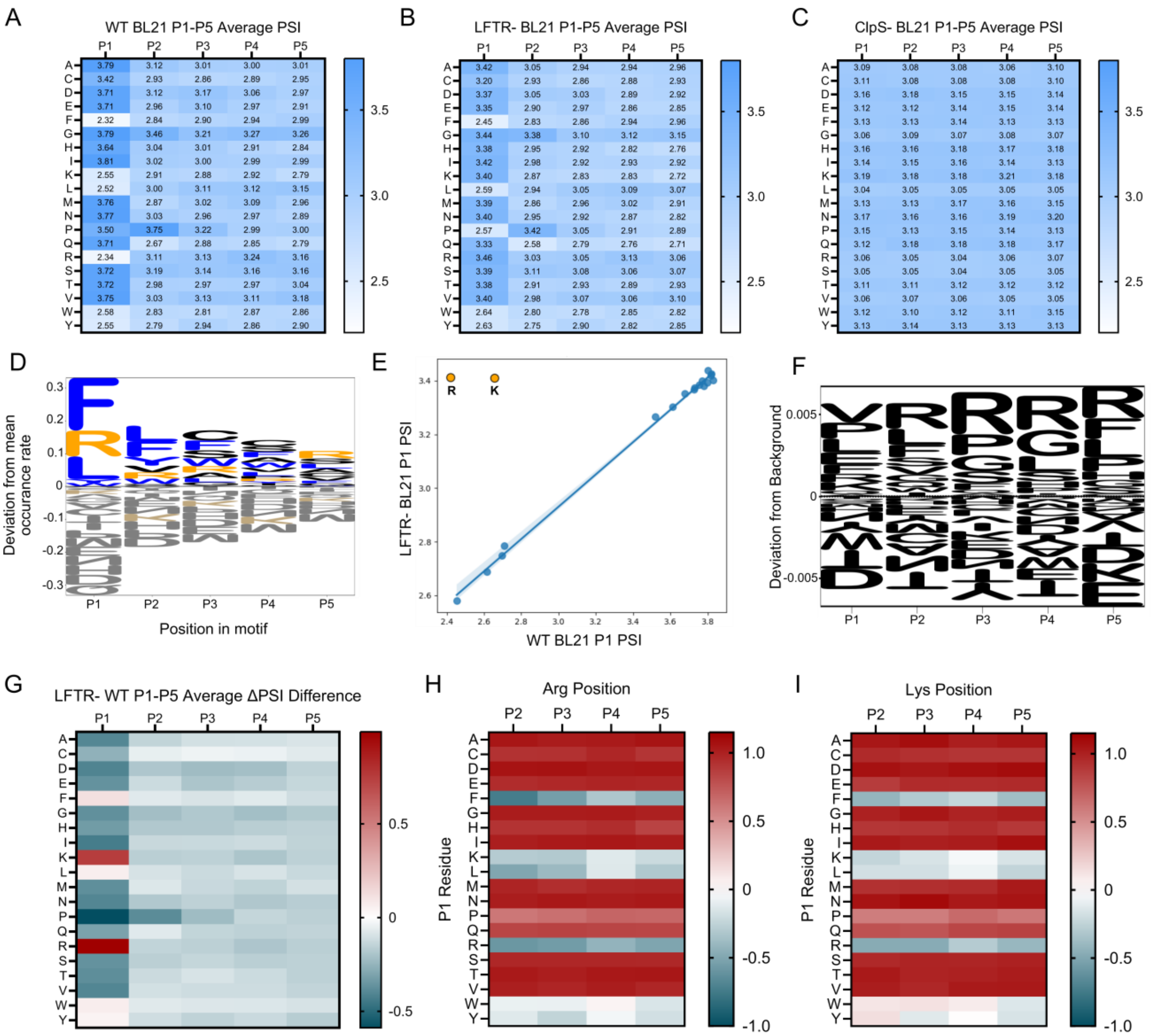

Supplemental Figure 2. A.) P1-P5 WT BL21 Heatmap A mean PSI heatmap generated from 2.29 million sequences for the first five positions of a protein in wild-type BL21. B.) P1-P5 LFTR- BL21 Heatmap A mean PSI heatmap generated from 2.34 million sequences for the first five positions of a protein in BL21 deficient in aat (LFTR). C.) P1-P5 ClpS- BL21 Heatmap A mean PSI heatmap generated from 2.19 million sequences for the first five positions of a protein in BL21 deficient in clpS. D.) Bulky and positively charged amino acids are enriched in low stability sequences. Weblogo depicting the difference in enrichment for each amino acid in each of the five positions in low PSI (PSI <2) samples compared to the entire dataset. E.) P1 amino acid correlation between WT BL21 and LFTR- BL21 sorts Correlation between PSI scores for N-terminal residues between

bioRxiv preprint doi: https://doi.org/10.1101/2025.05.22.655665; this version posted July 15, 2025. The copyright holder for this preprint (which was not certified by peer review) is the author/funder, who has granted bioRxiv a license to display the preprint in perpetuity. It is made available under aCC-BY 4.0 International license.

WT BL21 and LFTR- BL21 (r = 1.00) in the 10 million event sort. Arg/Lys/Pro were excluded from regression analysis. Arg and Lys separately highlighted in orange. Ubiquitin upstream of P1 Pro is not fully cleaved, complicating analysis. F.) Pro, Arg, Leu, and small amino acids are weakly enriched in low PSI sequences in the ClpS- dataset. Enrichment values of amino acids in sequences with a PSI <2 in the ClpS- dataset were subtracted from enrichment values from the whole dataset. Changes in representation cumulatively were <1% in either direction. G. P1 Arg and P1 Lys stability shifts are observed as a primary difference between LFTR- and WT datasets. Heatmap depicts the change in PSI for each AA-position combination between LFTR- and WT datasets. H. Internal Arg heatmaps per P1 residue. Heatmaps depict the average PSI for Arg at each position for any given P1 residue in the WT dataset. I. Internal Lys heatmaps per P1 residue. Heatmaps depict the average PSI for Lys at each position for any given P1 residue in the WT dataset.

bioRxiv preprint doi: https://doi.org/10.1101/2025.05.22.655665; this version posted July 15, 2025. The copyright holder for this preprint (which was not certified by peer review) is the author/funder, who has granted bioRxiv a license to display the preprint in perpetuity. It is made available under aCC-BY 4.0 International license.

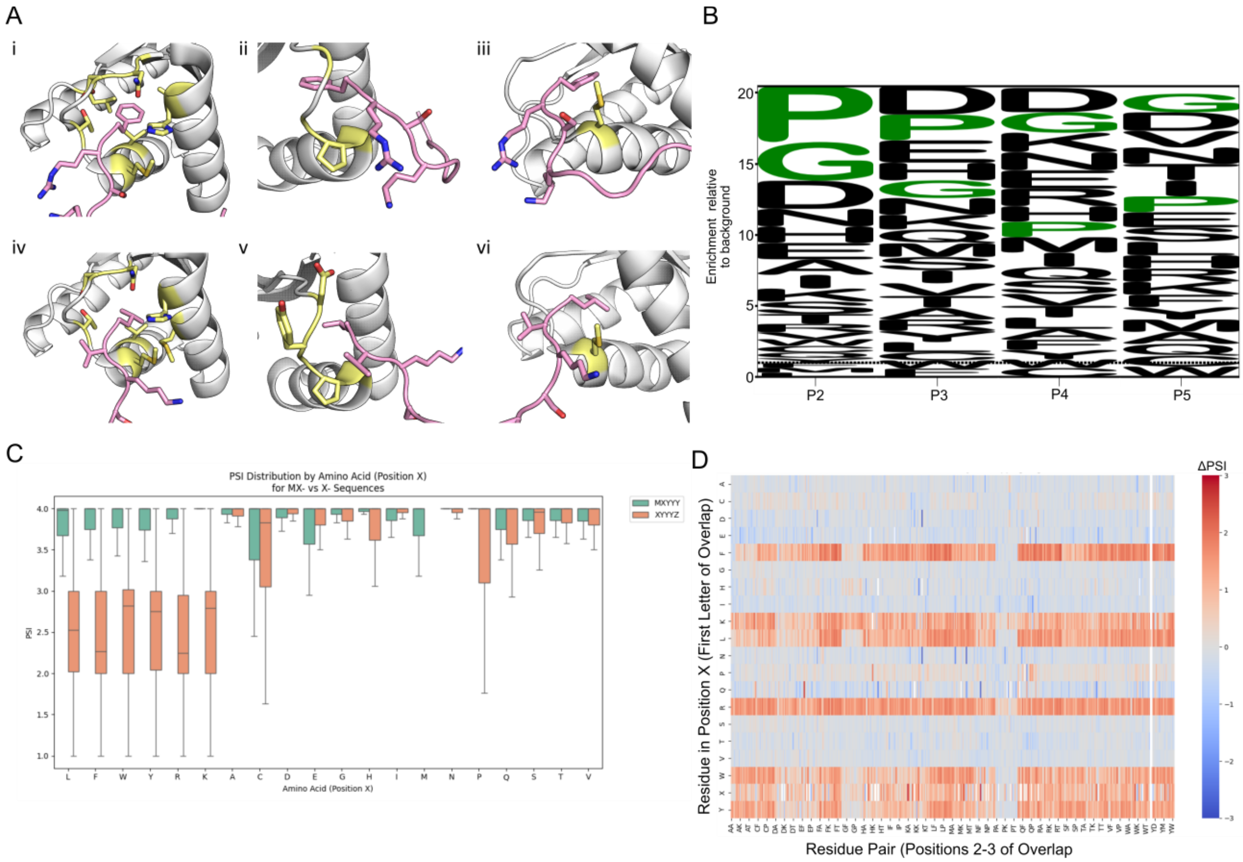

Supplemental Figure 3. A.) Structures of ClpS bound to peptide substrates provide limited insight into the molecular basis of substrate specificity downstream of P1. i-iii: Structures of ClpS bound to Fpep with nearby ClpS residues highlighted in yellow for (i) P1, (ii) P2, and (iii) P3. (PDB ID: 2WA8) iv-vi: Structures of ClpS bound to Lpep with nearby ClpS residues highlighted in yellow for (iv) P1, (v) P2, and (vi) P3. (PDB ID: 2W9R). No interacting residues within 4 A were obtained for P4 and P5 of the peptides. B.) Glycine and proline are highly enriched in high stability P1 FLWYRK sequences. A P2-P5 WebLogo showing high stability P1 FLWYRK (PSI >3) sequence enrichment relative to the entire wild-type dataset for each of the five positions studied. Glycine and proline highlighted in green. C.) MX- Motifs do not show similar PSI distributions for P1 LFWYRK motifs. Mean PSI boxplots for residues in position X from the 10M event sort. MXYYY is in green and XYYYZ is in orange. D.) Change in PSI between MXYYY and XYYYZ datasets, with the X-position on the y-axis and the first two Y positions in the x-axis.

bioRxiv preprint doi: https://doi.org/10.1101/2025.05.22.655665; this version posted July 15, 2025. The copyright holder for this preprint (which was not certified by peer review) is the author/funder, who has granted bioRxiv a license to display the preprint in perpetuity. It is made available under aCC-BY 4.0 International license.

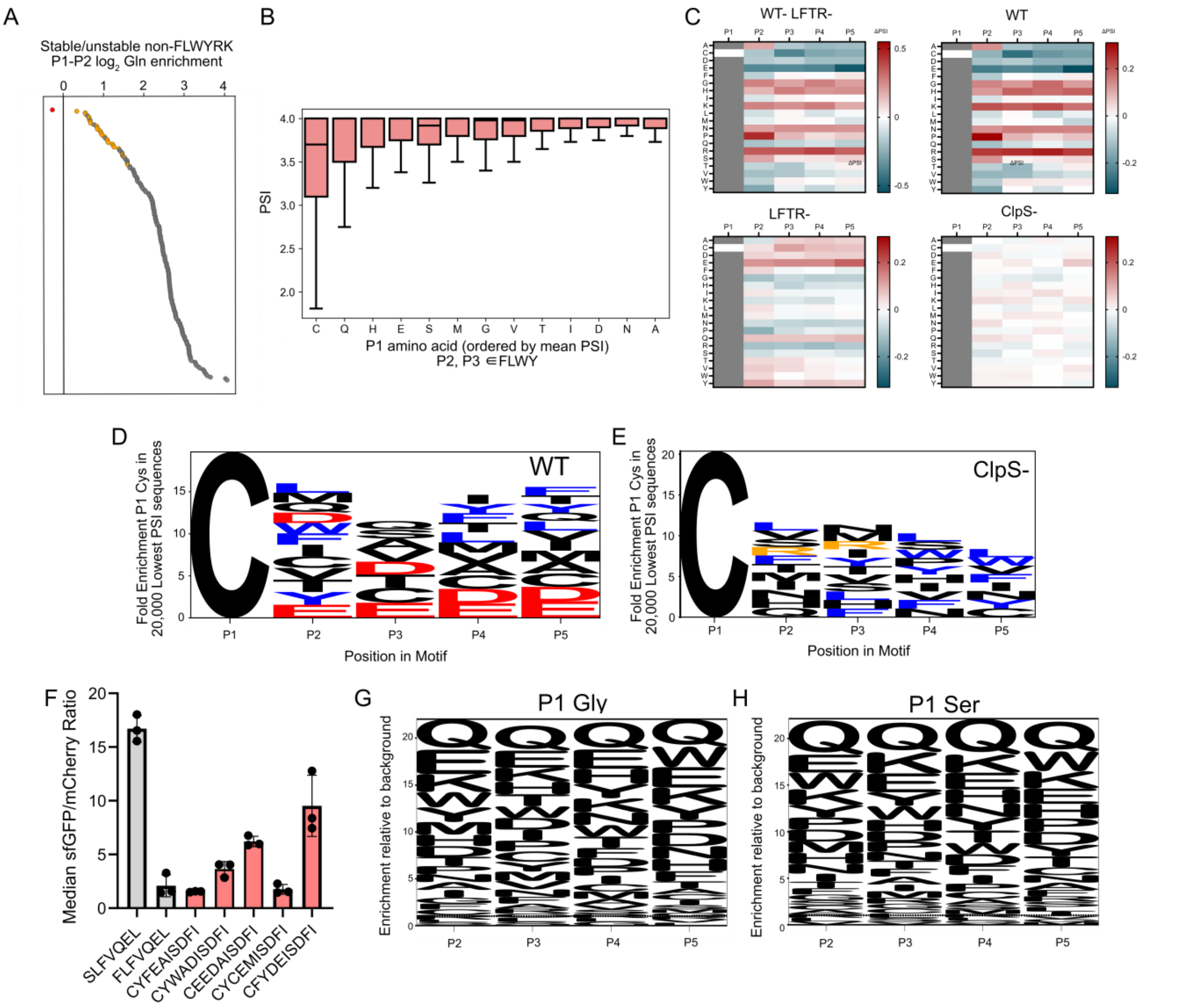

Supplemental Figure 4. A.) P1/P2 Gln is noticeably enriched in low stability P1-P2 motifs that do not have P1 FLWYRK. Log2 enrichment of P1-P2 motifs in low stability (PSI <2) motifs relative to high stability (PSI >3) motifs where FLWYRK are excluded from P1. Motifs with Gln in P1 or P2 are in orange, QQ is in red. B.) P1 Cys is the most destabilizing non FLWYRK residue when P2 and P3 are bulky. Boxplot showing PSI distributions for non-P1 FLWYRKP motifs that have P2 and P3 residues in FLWY. C.) P1 Cys mean PSI heatmaps in various backgrounds reveal P2-P5 charge and bulky residue preferences. Sequences with P1 Cys were filtered in each dataset. Data represents differences from the P1 Cys global mean. WT- LFTR- represents the subtraction of difference from the mean values between the WT and LFTR- datasets. D.) Low stability P1 Cys sequences are rich in negatively charged and bulky amino acids at P2-P5. Weblogo depicting enriched amino acids for

bioRxiv preprint doi: https://doi.org/10.1101/2025.05.22.655665; this version posted July 15, 2025. The copyright holder for this preprint (which was not certified by peer review) is the author/funder, who has granted bioRxiv a license to display the preprint in perpetuity. It is made available under aCC-BY 4.0 International license.

the 20,000 lowest PSI motifs with P1 Cys in the WT dataset. Bulky residues (FLWY) depicted in blue and negatively charged residues (DE) depicted in red. E.) Negatively charged residues are absent in low stability P1 Cys sequences. Weblogo depicting enriched amino acids for the 20,000 lowest PSI motifs with P1 Cys in the ClpS- dataset. Bulky residues (FLWY) depicted in blue and postively charged residues (RK) depicted in orange. F.) Full panel P1 Cys screen reveals putative N-degrons. Flow cytometry data collected from biological triplicates for the full panel of Nt-Cys screened sequences. G.) Enriched residues in low stability sequences for P1 Gly. Weblogo depicting the enrichment of amino acids relative to the whole population for the lowest 20,000 PSI sequences with P1 Gly. H.) Enriched residues in low stability sequences for P1 Ser. Weblogo depicting the enrichment of amino acids relative to the whole population for the lowest 20,000 PSI sequences with P1 Ser.

bioRxiv preprint doi: https://doi.org/10.1101/2025.05.22.655665; this version posted July 15, 2025. The copyright holder for this preprint (which was not certified by peer review) is the author/funder, who has granted bioRxiv a license to display the preprint in perpetuity. It is made available under aCC-BY 4.0 International license.

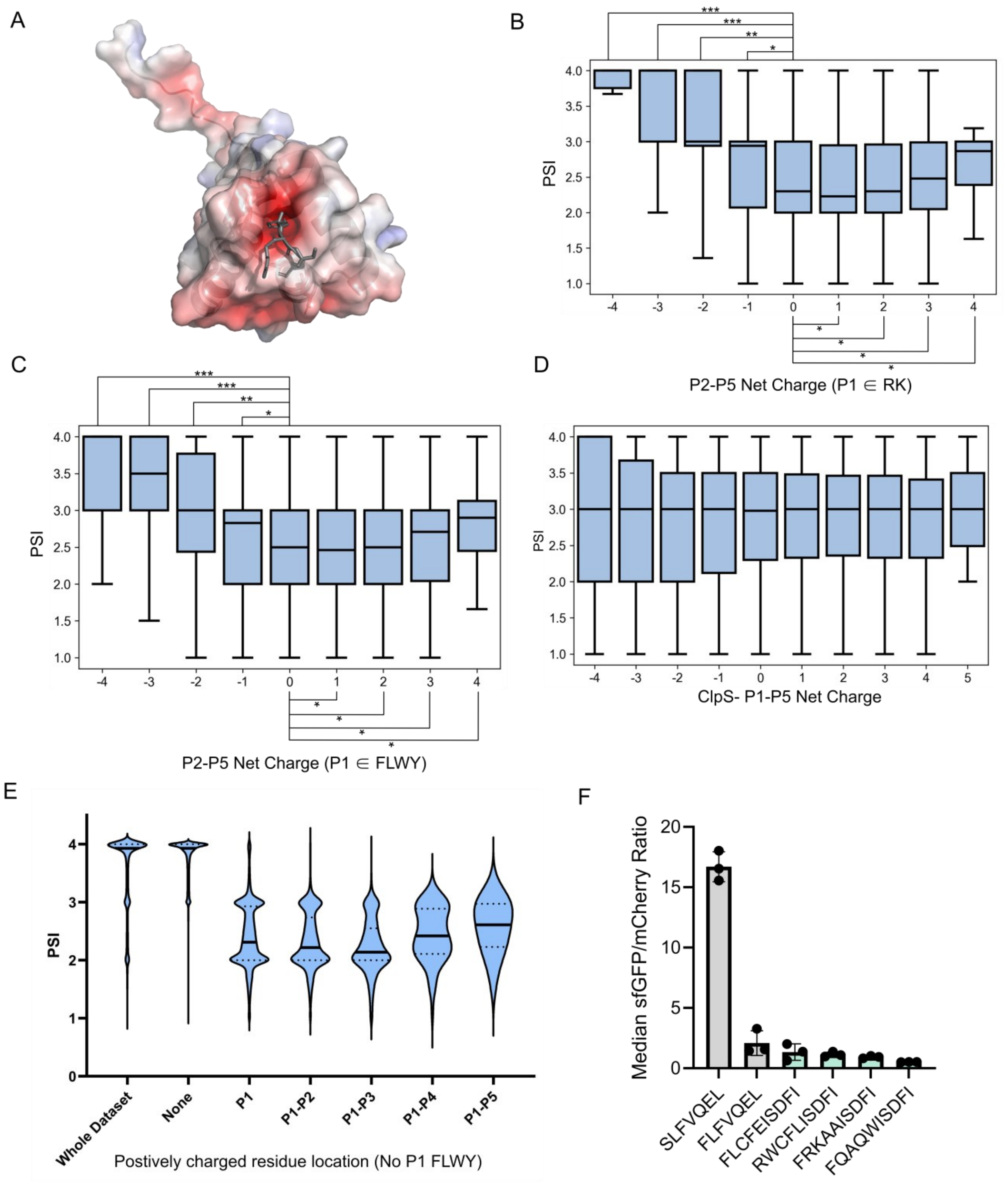

Supplemental Figure 5. A.) The ClpS binding pocket has negative electrostatic potential. ClpS bound to a substrate (PDB 2WA8) visualized using the APBS Pymol plugin to create a surface map

bioRxiv preprint doi: https://doi.org/10.1101/2025.05.22.655665; this version posted July 15, 2025. The copyright holder for this preprint (which was not certified by peer review) is the author/funder, who has granted bioRxiv a license to display the preprint in perpetuity. It is made available under aCC-BY 4.0 International license.

where regions of negative electrostatic potential are highlighted in red and regions of positive electrostatic potential are highlighted in blue. B.) Separation of charged and bulky P1 residue net charge heatmaps. C.) ClpS- sequences show charge parity. P1-P5 net charge heatmap for data collected in ClpS- Bl21. D.) Multiple positively charged residues at and near P1 lead to low PSI sequences. Violin plot depicting the PSI distribution in wild-type BL21 for sequences with one or more RK residues in series at and adjacent to the N-terminus. E.) Sequences with multiple FLWYRK(Q) residues show low stability. Flow cytometry data from the protein stability assay depicting sequences carrying multiple FLWYRK or P2 Gln residues exhibited low sfGFP:mCherry ratios relative to literature controls. All p-values in comparison to 0 P2-P5 Gly/Ser show $p < 0.05$. p-values evaluated using a Mann-Whitney U test, effect size magnitude (ES) ranges from 0 to 1, as evaluated using Cliff's delta. * = $p < 0.05$, ES 0-0.25; ** = $p < 0.05$, ES 0.25-0.50, *** = $p < 0.05$, ES 0.50-0.75, **** = $p < 0.05$, ES 0.75-1.0

bioRxiv preprint doi: https://doi.org/10.1101/2025.05.22.655665; this version posted July 15, 2025. The copyright holder for this preprint (which was not certified by peer review) is the author/funder, who has granted bioRxiv a license to display the preprint in perpetuity. It is made available under aCC-BY 4.0 International license.

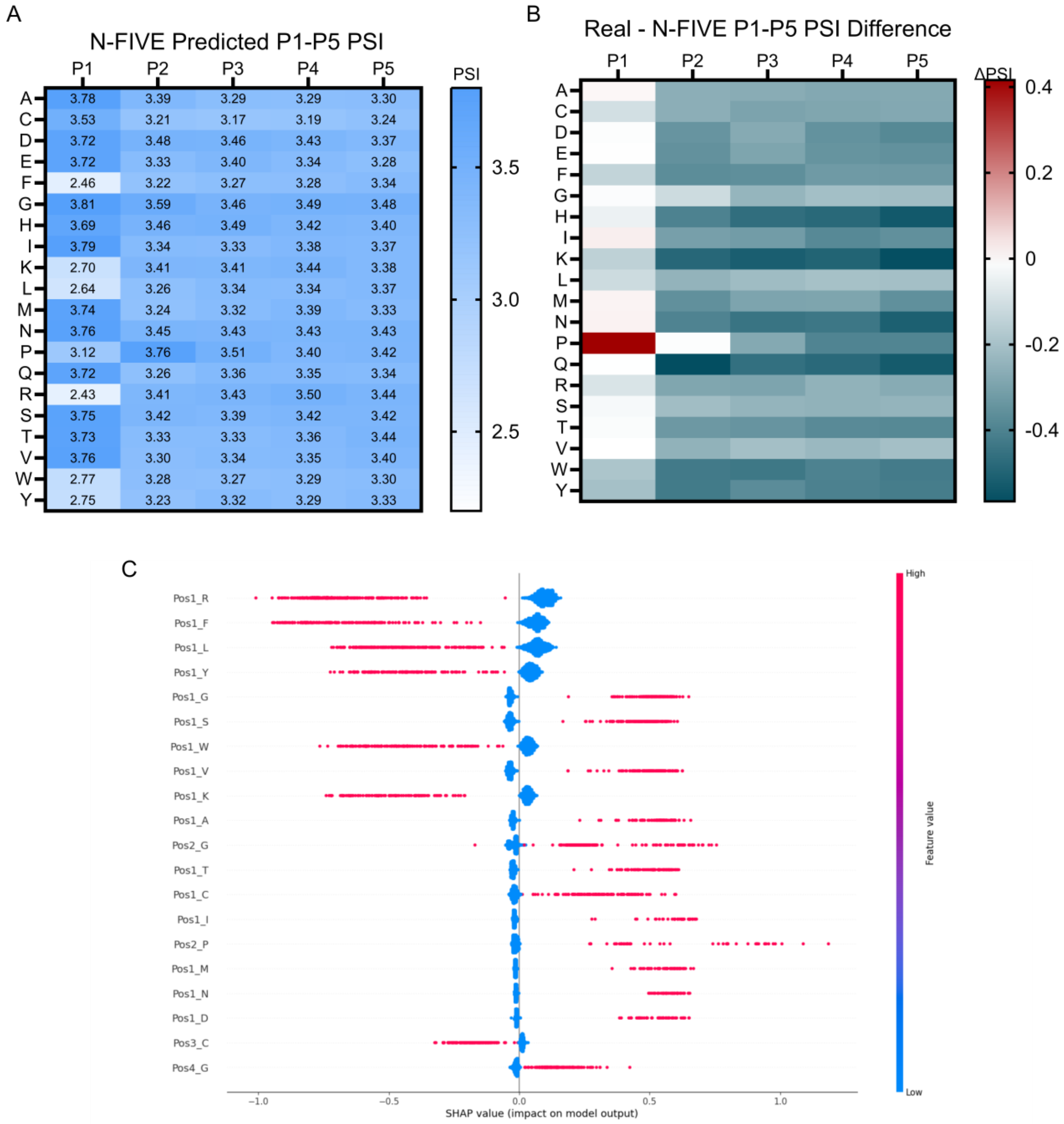

Supplemental Figure 6. A.) N-FIVE PSI predictions for the mean PSI heatmap for all 3.2 million sequences in wild-type *Escherichia coli* BL21. B.) Change in PSI for each cell between the collected WT BL21 dataset and the N-FIVE predicted dataset. C.) Individual matrix components for N-FIVE predictions. SHAP values showing the contribution of the 20 most impactful matrix features onto the N-FIVE model.

bioRxiv preprint doi: https://doi.org/10.1101/2025.05.22.655665; this version posted July 15, 2025. The copyright holder for this preprint (which was not certified by peer review) is the author/funder, who has granted bioRxiv a license to display the preprint in perpetuity. It is made available under aCC-BY 4.0 International license.

Supplemental Table 1. Important gene sequences

Genetic construct	DNA Sequence
Dual fluorescent reporter (mCherry-Ub-RCKGLAALPHS-sfGFP-His6x	ATGGTGAGCAAGGGCGAGGaggataacatggccatcatcaaggagt<br>tcatgcgcttcaaggtgcacatggagggctccgtgaacggccacgagttcgagat<br>cgagggcgagggcgagggccgcccctacgagggcacccagaccgccaagctg<br>aaggtgaccaagggtggcccctgcccttcgcctgggacatcctgtcccctcag<br>ttcatgtacggctccaaggcctacgtgaagcaccccgccgacatccccgacta<br>cttgaagctgtccttccccgagggcttcaagtgggagcgcgtgatgaacttcgagg<br>acggcggcgtggtgaccgtgacccaggactcctcactgcaggacggcgagttca<br>tctacaaggtgaagctgcgcggcaccaacttcccctccgacggccccgtaatg<br>cagaagaagaccatgggctgggaggcctcctccgagcggatgtaccccgagga<br>cggcgccctgaagggcgagatcaagcagaggctgaagctgaaggacggcggc<br>cactacgacgctgaggtcaagaccacctacaaggccaagaagcccgtgcagc<br>tgcccggcgcctacaacgtcaacatcaagttggacatcacctcccacaacgag<br>gactacaccatcgtggaacagtacgaacgcgctgagggccgccactccaccg<br>gcggcATGGACGAGCTGTACAAGCAGATTTTTGTGAAGACTT<br>TAACAGGTAAGACGATTACCCTGGAGGTGGAGTCCTCGG<br>ACACCATCGATAATGTAAAATCAAAAATCCAAGATAAGGAA<br>GGAATCCCTCCAGACCAGCAACGTCTGATTTTCGCAGGTA<br>AACAACTGGAGGATGGTCGCACGCTTTCGGACTACAACAT<br>CCAGAAAGAATCTACCCTTCATTTGGTTCTGCGTCTGCGTG<br>GAGGACGCTGCAAAGGACTTGCAGCACTGCCCCATTCCg<br>catccaagggcgaggagctctttactggcgtagtaccaattctcgtagagctcgat<br>ggcgatgtaaatggccataagttttccgtacgcggcgagggcgagggcgatgcaa<br>ctaacggcaagctcactctcaagtttatttgtactactggcaagctcccagtacc<br>atggccaactctcgtaactactctgacctatggcgtacaatgtttttcccgctatc<br>cagatcacatgaagcaacatgatttttttaagtccgcaatgccagagggctatgta<br>caagagcgcactattagctttaaggatgatggcacctataagactcgcgcagag<br>gtaaagtttgagggcgatactctcgtaaatcgcattgagctcaagggcattgatttt<br>aaggaggatggcaatattctcggccataagctggagtataatttcaattcccataa<br>tgtatacattaccgcagataagcaaaagaatggcattaaggcgaattttaagattc<br>gccataatgtggaggatggctccgtacaactcgcagatcattatcaacaaaata<br>ctccaattggcgatggcccagtactcctcccagataatcattatctctccactca<br>atccgtgctctccaaagatccaaatgagaagcgcgatcacatggtactcctgga<br>gtttgtaactgcagcaggcattactcatggcatggatgagctctataagctcgagc<br>accaccaccaccaccactaa

bioRxiv preprint doi: https://doi.org/10.1101/2025.05.22.655665; this version posted July 15, 2025. The copyright holder for this preprint (which was not certified by peer review) is the author/funder, who has granted bioRxiv a license to display the preprint in perpetuity. It is made available under aCC-BY 4.0 International license.

scUbp1, truncated	ATGGGGAGTGGGTCTTTCATTGCTGGGCTTGTCAACGATG<br>GTAATACGTGTTTTATGAACTCGGTTCTTCAGTCCCTTGCTA<br>GTAGCCGTGAACTTATGGAGTTTTTGGATAATAATGTAATCC<br>GTACATATGAAGAAATTGAACAGAACGAGCACAATGAGGA<br>AGGTAATGGCCAAGAGAGCGCACAAGATGAGGCAACTCA<br>CAAAAAAAACACTCGCAAGGGAGGTAAGGTCTATGGGAA<br>GCATAAAAAGAAATTAAACCGCAAATCTTCTAGCAAGGAA<br>GACGAAGAAAAGTCGCAAGAACCAGACATTACGTTTTCGG<br>TGGCGTTGCGTGATCTGCTGAGCGCATTAAATGCTAAGTAT<br>TATCGCGACAAACCCTACTTTAAGACTAACTCTTTATTAAAA<br>GCGATGAGCAAGTCCCCGCGCAAAAATATCTTGCTTGGGT<br>ACGATCAAGAAGACGCTCAGGAATTTTTTCAAAACATTCTT<br>GCGGAGTTAGAATCTAATGTCAAGTCGTTAAACACAGAAA<br>AGCTTGATACTACACCGGTAGCCAAGTCCGAACTTCCAGA<br>CGATGCTCTGGTTGGCCAATTAAACCTTGGTGAGGTAGGC<br>ACCGTGTACATTCCCACAGAACAAATTGACCCCAATTCGA<br>TTTTACATGACAAATCGATTCAAAACTTTACCCCCTTTAAAC<br>TGATGACCCCGTTGGATGGGATCACGGCTGAGCGCATCG<br>GCTGCCTGCAATGCGGAGAGAACGGGGGAATTCGCTACA<br>GTGTTTTCAGCGGATTAAGTTTGAACCTGCCGAATGAAAAT<br>ATTGGAAGCACTCTTAAACTGTCCCAGTTACTGTCCGATTG<br>GTCGAAACCCGAGATTATCGAGGGTGTTGAATGCAACCGT<br>TGCGCTTTAACAGCTGCGCACTCACACTTGTTTGGCCAATT<br>AAAGGAGTTTGAGAAGAAACCTGAAGGCTCGATTCCCGAA<br>AAACTTATTAATGCCGTAAAGGACCGCGTGCACCAGATCG<br>AAGAGGTCTTGGCAAAGCCGGTTATCGACGATGAAGATTA<br>TAAAAAATTGCATACTGCGAATATGGTCCGCAAGTGTTCAA<br>AAAGTAAACAAATTCTTATCTCTCGTCCACCACCTTTGTTGT<br>CTATTCATATCAACCGCTCTGTTTTCGACCCGCGCACCTA<br>CATGATTCGCAAGAACAACTCCAAGGTTTTGTTCAAGTCAC<br>GCTTGAACCTGGCACCCTGGTGCTGTGATATCAACGAAAT<br>CAATCTTGACGCACGCCTTCCGATGTCGAAGAAGGAAAAA<br>GCAGCTCAACAAGATTCTTCTGAAGACGAGAACATTGGCG<br>GAGAGTACTATACTAAATTGCATGAACGTTTTGAGCAGGAG<br>TTTGAAGATTCTGAAGAAGAGAAGGAATACGATGATGCAG<br>AGGGTAATTATGCATCGCATTATAACCATACCAAGGACATC<br>TCCAACTACGATCCATTGAATGGAGAAGTCGACGGTGTGA<br>CTTCCGATGATGAGGATGAATACATTGAAGAGACAGACGC<br>GTTGGGGAATACCATCAAAAAACGTATTATTGAACACTCCG<br>ACGTGGAGAACGAAAACGTGAAGGATAATGAAGAACTTCA<br>GGAGATCGATAACGTTAGCTTGGATGAGCCAAAAATTAAT<br>GTCGAGGACCAGCTTGAAACGAGTTCTGATGAGGAAGAC<br>GTTATTCCTGCTCCACCCATCAACTACGCTCGCAGCTTTA<br>GTACGGTCCCAGCGACCCCTTTAACTTACTCTTTGCGCAG

bioRxiv preprint doi: https://doi.org/10.1101/2025.05.22.655665; this version posted July 15, 2025. The copyright holder for this preprint (which was not certified by peer review) is the author/funder, who has granted bioRxiv a license to display the preprint in perpetuity. It is made available under aCC-BY 4.0 International license.

	CGTCATCGTGCACTATGGGACTCACAACTACGGACATTAT ATTGCATTTCGCAAGTATCGTGGATGTTGGTGGCGCATCTC CGATGAGACGGTCTATGTGGTAGATGAGGCCGAAGTACTG TCAACACCGGGGGTATTTATGCTTTTCTACGAGTATGATTT CGACGAGGAGACCGGAAAAATGAAAGACGACTTAGAAGC TATCCAGAGCAATAATGAGGAAGATGACGAGAAAGAACAG GAACAGAAGGGTGTCCAGGAGCCAAAAGAATCCCAGGAG CAAGGCGAAGGCGAAGAACAAGAAGAAGGGCAAGAGCA AATGAAATTTGAGCGTACGGAGGATCATCGCGACATTTCA GGGAAGGATGTGAATTAA

Supplemental Table 2. Important oligos

Oligos marked with SEQ were used for Sanger sequencing. Neo-N-terminus (NT) cloning featured a target NT primer + oSS196 as piece 1 and oSS195 + oSS055 as piece 2 used to generate amplicons from a pEVOL dual reporter plasmid.

Oligo ID	Sequence	Purpose
	Sequencing oligos	
oSS045 SEQ REV	tctagcacgcgtaccatg	sfGFP Sanger sequencing
oSS083 SEQ FWD	Cacggcagaaaagtccac	mCherry Sanger sequencing
oSS084 SEQ FWD	CCAGCAACGTCTGATTTTC	Neo-Nt Sanger sequencing
oSS264 SEQ FWD	caacgaggactacaccatcg	mCherry/Ub Sanger sequencing
	Neo-N-terminus insert cloning oligos	
oSS155 FWD	TTCTGCGTCTGCGTGGAGGACGTTGCGG GGGTGCTATCATCAGTGACTTCATCgcatcc aagggcGCGG	ArgRAP2.2 NT cloning
oSS199 FWD	TTCTGCGTCTGCGTGGAGGAAGCCTGTTT GTGCAGGAACTGgcatccaagggcgaggag	SLFVQEL NT cloning
oSS200 FWD	TTCTGCGTCTGCGTGGAGGATTTCTGTTTG TGCAGGAACTGgcatccaagggcgaggag	FLFVQEL NT cloning
oSS262 FWD	TGGTTCTGCGTCTGCGTGGAGGATTGGTG AAGACCAAGGCGTCCAATTTATTAgcatcca agggcgaggag	DPS NT cloning
oSS388 FWD	TTCTGCGTCTGCGTGGAGGAGATCATGGA TCGGGTGCCTGGCTGTTGCCGgcatccaagg gcgaggag	D-Bgal NT cloning

bioRxiv preprint doi: https://doi.org/10.1101/2025.05.22.655665; this version posted July 15, 2025. The copyright holder for this preprint (which was not certified by peer review) is the author/funder, who has granted bioRxiv a license to display the preprint in perpetuity. It is made available under aCC-BY 4.0 International license.

oSS698 FWD	TTCTGCGTCTGCGTGGAGGANNKNNKNN KNNKNNKATCAGTGACTTCATCgcatccaag	P1-P5 NT library cloning
oSS1035 FWD	TTCTGCGTCTGCGTGGAGGATGTTTTTACG AAGCTATCAGTGACTTCATCgcatccaag	CFYEA NT FWD
oSS1036 FWD	TTCTGCGTCTGCGTGGAGGATGCTATTGG GCCGACATCAGTGACTTCATCgcatccaag	CYWAD NT FWD
oSS1037 FWD	TTCTGCGTCTGCGTGGAGGATGCGAGGA GGACGCAATCAGTGACTTCATCgcatccaag	CEEDA NT FWD
oSS1038 FWD	TTCTGCGTCTGCGTGGAGGATGTTACTGT GAGATGATCAGTGACTTCATCgcatccaag	CYCEM NT FWD
oSS1039 FWD	TTCTGCGTCTGCGTGGAGGATGTGACTAC GACGAGATCAGTGACTTCATCgcatccaag	CFYDE NT FWD
oSS1040 FWD	TTCTGCGTCTGCGTGGAGGACCGTACTTT GACGAGATCAGTGACTTCATCgcatccaag	PYDFE NT FWD
oSS1041 FWD	TTCTGCGTCTGCGTGGAGGAATTTTTTTTG CAGAGATCAGTGACTTCATCgcatccaag	IFFAE NT FWD
oSS1042 FWD	TTCTGCGTCTGCGTGGAGGAATTTGGTAT AAGCATATCAGTGACTTCATCgcatccaag	IWYKH NT FWD
oSS1043 FWD	TTCTGCGTCTGCGTGGAGGATTTCAGAAG CAAAAAATCAGTGACTTCATCgcatccaag	FQKQK NT FWD
oSS1044 FWD	TTCTGCGTCTGCGTGGAGGACGTCAATGG TATCACATCAGTGACTTCATCgcatccaag	RQWYH NT FWD
oSS1045 FWD	TTCTGCGTCTGCGTGGAGGATTTGACGAG GATGAAATCAGTGACTTCATCgcatccaag	FDEDE NT FWD
oSS1046 FWD	TTCTGCGTCTGCGTGGAGGATTCGATGAG GCGGCCATCAGTGACTTCATCgcatccaag	FDEAA NT FWD
oSS1047 FWD	TTCTGCGTCTGCGTGGAGGATTTGCAGCC GCAGCCATCAGTGACTTCATCgcatccaag	FAAAA NT FWD
oSS1048 FWD	TTCTGCGTCTGCGTGGAGGATTTCGCAAG GCAGCTATCAGTGACTTCATCgcatccaag	FRKAA NT FWD
oSS1050 FWD	TTCTGCGTCTGCGTGGAGGAGGATTGGTG CGTGGTATCAGTGACTTCATCgcatccaag	GLVRG NT FWD

bioRxiv preprint doi: https://doi.org/10.1101/2025.05.22.655665; this version posted July 15, 2025. The copyright holder for this preprint (which was not certified by peer review) is the author/funder, who has granted bioRxiv a license to display the preprint in perpetuity. It is made available under aCC-BY 4.0 International license.

oSS1051 FWD	TTCTGCGTCTGCGTGGAGGAGGTTTGCGC TTACGCATCAGTGACTTCATCgcatccaag	GLRLR NT FWD
oSS1052 FWD	TTCTGCGTCTGCGTGGAGGATTGGTGCGC CTGGGGATCAGTGACTTCATCgcatccaag	LVRLG NT FWD
oSS1053 FWD	TTCTGCGTCTGCGTGGAGGAGGCAGTGTT CTTCGTATCAGTGACTTCATCgcatccaag	GSVLR NT FWD
oSS1054 FWD	TTCTGCGTCTGCGTGGAGGACACTGGATG CAAAAAATCAGTGACTTCATCgcatccaag	HWMQK NT FWD
oSS1055 FWD	TTCTGCGTCTGCGTGGAGGAAACATTCAG ATGAAAATCAGTGACTTCATCgcatccaag	NIQMK NT FWD
oSS1056 FWD	TTCTGCGTCTGCGTGGAGGATCATTGTGG AAATACATCAGTGACTTCATCgcatccaag	SLWKY NT FWD
oSS1057 FWD	TTCTGCGTCTGCGTGGAGGATCTTACATG ATGGATATCAGTGACTTCATCgcatccaag	SYMMD NT FWD
oSS1058 FWD	TTCTGCGTCTGCGTGGAGGAATCAAGAAA ACGCAGATCAGTGACTTCATCgcatccaag	IKKTQ NT FWD
oSS1059 FWD	TTCTGCGTCTGCGTGGAGGACTTGCGGC CAGCTACATCAGTGACTTCATCgcatccaag	LAASY NT FWD
oSS1060 FWD	TTCTGCGTCTGCGTGGAGGACTTGACCGT AATAGCATCAGTGACTTCATCgcatccaag	LDRNS NT FWD
oSS1061 FWD	TTCTGCGTCTGCGTGGAGGATTAGCCGTC CCGCGTATCAGTGACTTCATCgcatccaag	LAVPR NT FWD
oSS1062 FWD	TTCTGCGTCTGCGTGGAGGATTAGGCCC CCGTTCAATCAGTGACTTCATCgcatccaag	LRPRS NT FWD
oSS1063 FWD	TTCTGCGTCTGCGTGGAGGATTAGGGGAT TCAGTCATCAGTGACTTCATCgcatccaag	LGDSV NT FWD
oSS1064 FWD	TTCTGCGTCTGCGTGGAGGACAGCAGTGT AAATGGATCAGTGACTTCATCgcatccaag	QQCKW NT FWD
oSS1065 FWD	TTCTGCGTCTGCGTGGAGGACATCAGGC CCAGTGGATCAGTGACTTCATCgcatccaag	HQAQW NT FWD

bioRxiv preprint doi: https://doi.org/10.1101/2025.05.22.655665; this version posted July 15, 2025. The copyright holder for this preprint (which was not certified by peer review) is the author/funder, who has granted bioRxiv a license to display the preprint in perpetuity. It is made available under aCC-BY 4.0 International license.

oSS1066 FWD	TTCTGCGTCTGCGTGGAGGATTCCAAGCGCAATGGATCAGTGACTTCATCgcatccaag	FQAQW NT FWD
oSS1067 FWD	TTCTGCGTCTGCGTGGAGGATGGGGAGCAGCAGTGATCAGTGACTTCATCgcatccaag	WGGAV NT FWD
oSS1068 FWD	TTCTGCGTCTGCGTGGAGGACGTGGGGCCGCCGTTATCAGTGACTTCATCgcatccaag	RGAAV NT FWD
oSS1069 FWD	TTCTGCGTCTGCGTGGAGGACTGGGCGCAGCAGTTATCAGTGACTTCATCgcatccaag	LGAAV NT FWD
oSS1070 FWD	TTCTGCGTCTGCGTGGAGGATTTTTGTGTTTTGAAATCAGTGACTTCATCgcatccaag	FLCFE NT FWD
oSS1071 FWD	TTCTGCGTCTGCGTGGAGGACGCTGGTGCTTCTTGATCAGTGACTTCATCgcatccaag	RWCFL NT FWD
Other cloning oligos		
oSS055 REV	TCCTCCACGCAGACGCAG	NT cloning
oSS195 FWD	gagtgtatactggcttactatgttggca	pEVOL mid BB cloning
oSS196 REV	tgccaacatagtaagccagtatacactc	pEVOL mid BB cloning

bioRxiv preprint doi: https://doi.org/10.1101/2025.05.22.655665; this version posted July 15, 2025. The copyright holder for this preprint (which was not certified by peer review) is the author/funder, who has granted bioRxiv a license to display the preprint in perpetuity. It is made available under aCC-BY 4.0 International license.

Supplemental File 2. Uncropped Western blots

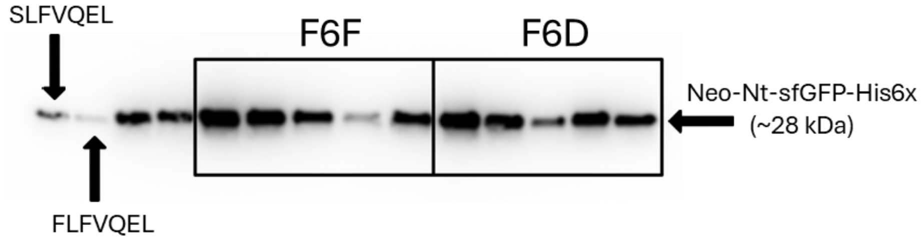

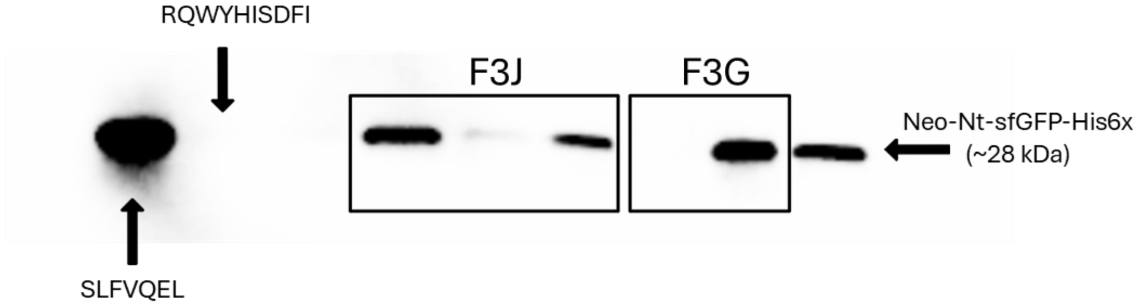

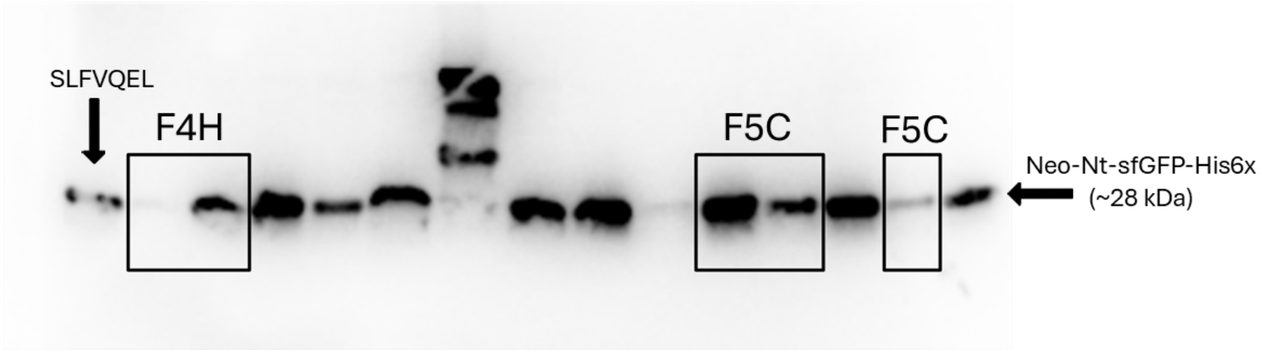

A consecutive lane was skipped in Figure 5C due to compounding factors that may promote stability for a neutrally charged motif, such as the presence of repetitive small amino acids at P2-P5 (FAAAISDFI).
