# Supplementary material for: Combinatorial mutagenesis of N-terminal sequences reveals unexpected and expanded stability determinants of the Escherichia coli N-degron pathway
Source: bioRxiv. 2025 May 22:2025.05.22.655665. Preprint. [Version 1] doi: 10.1101/2025.05.22.655665 (PMC12258705; doi:10.1101/2025.05.22.655665)
Supplement: Supplementary file 1 [file NIHPP2025.05.22.655665v1-supplement-1.pdf]

bioRxiv preprint doi: https://doi.org/10.1101/2025.05.22.655665; this version posted May 22, 2025. The copyright holder for this preprint (which was not certified by peer review) is the author/funder, who has granted bioRxiv a license to display the preprint in perpetuity. It is made available under aCC-BY 4.0 International license.

Supplementary Materials

Supplemental Figures

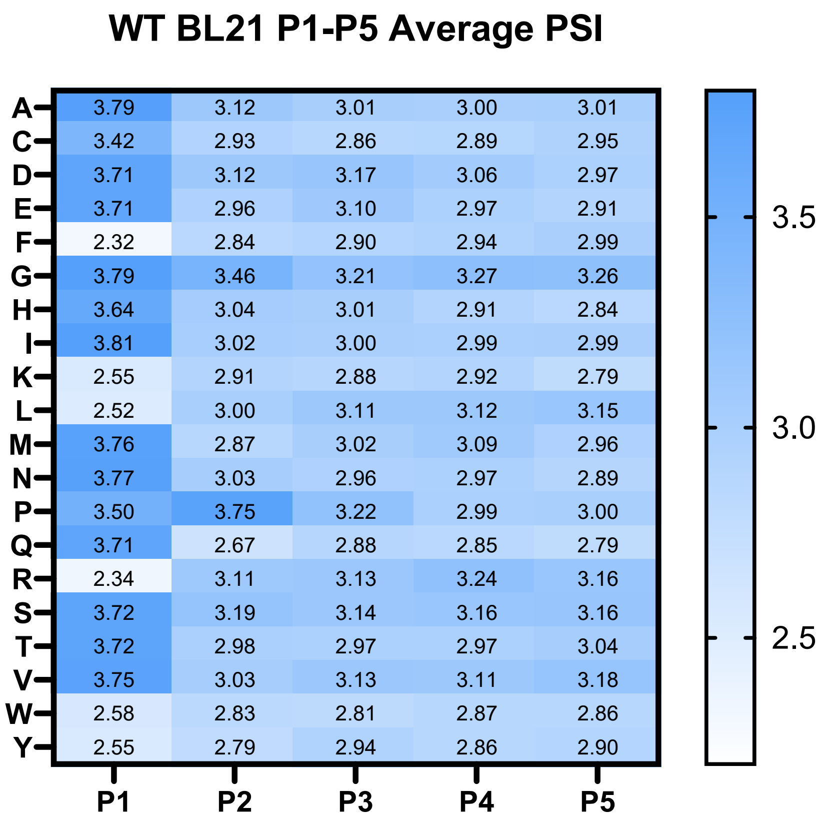

Supplemental Figure 1. P1-P5 WT BL21 Heatmap A mean PSI heatmap generated from 2.29 million sequences for the first five positions of a protein in wild-type BL21.

bioRxiv preprint doi: https://doi.org/10.1101/2025.05.22.655665; this version posted May 22, 2025. The copyright holder for this preprint (which was not certified by peer review) is the author/funder, who has granted bioRxiv a license to display the preprint in perpetuity. It is made available under aCC-BY 4.0 International license.

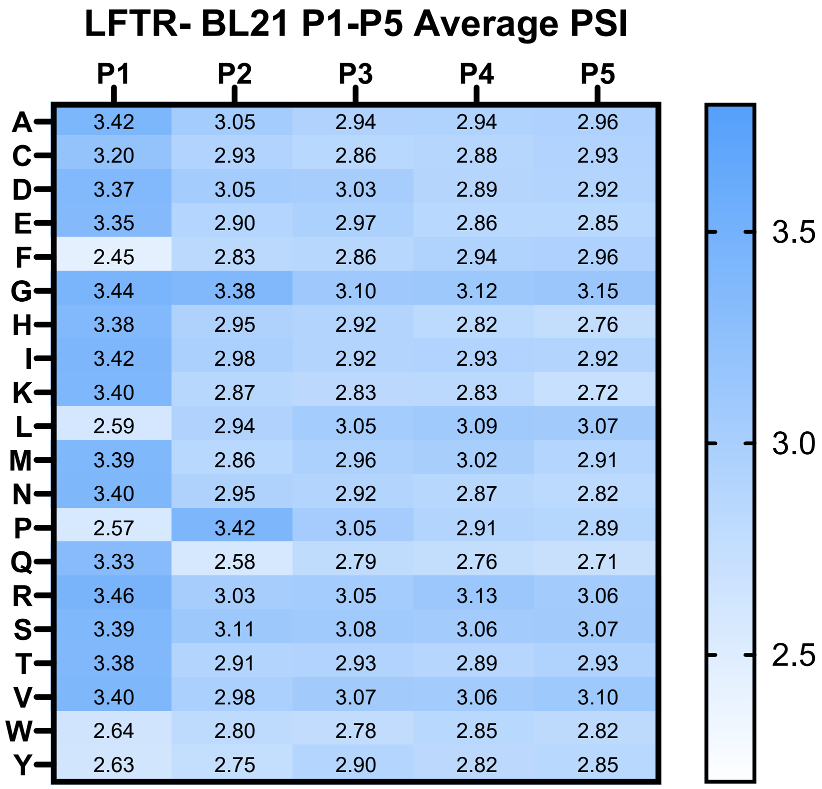

Supplemental Figure 2. P1-P5 LFTR- BL21 Heatmap A mean PSI heatmap generated from 2.34 million sequences for the first five positions of a protein in BL21 deficient in *aat* (LFTR).

bioRxiv preprint doi: https://doi.org/10.1101/2025.05.22.655665; this version posted May 22, 2025. The copyright holder for this preprint (which was not certified by peer review) is the author/funder, who has granted bioRxiv a license to display the preprint in perpetuity. It is made available under aCC-BY 4.0 International license.

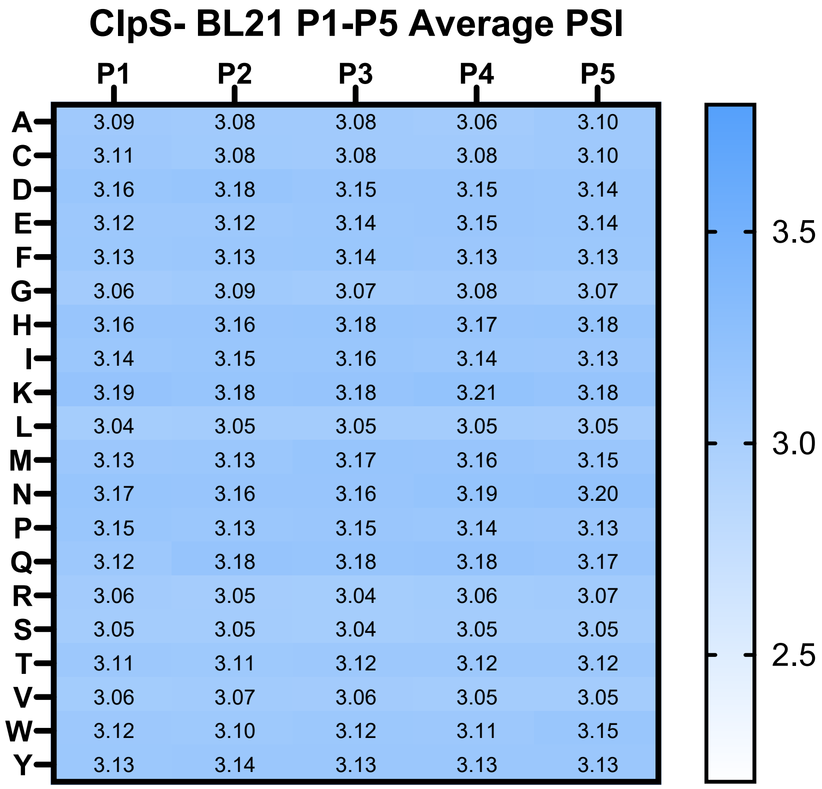

Supplemental Figure 3. P1-P5 ClpS- BL21 Heatmap A mean PSI heatmap generated from 2.19 million sequences for the first five positions of a protein in BL21 deficient in *clpS*.

bioRxiv preprint doi: https://doi.org/10.1101/2025.05.22.655665; this version posted May 22, 2025. The copyright holder for this preprint (which was not certified by peer review) is the author/funder, who has granted bioRxiv a license to display the preprint in perpetuity. It is made available under aCC-BY 4.0 International license.

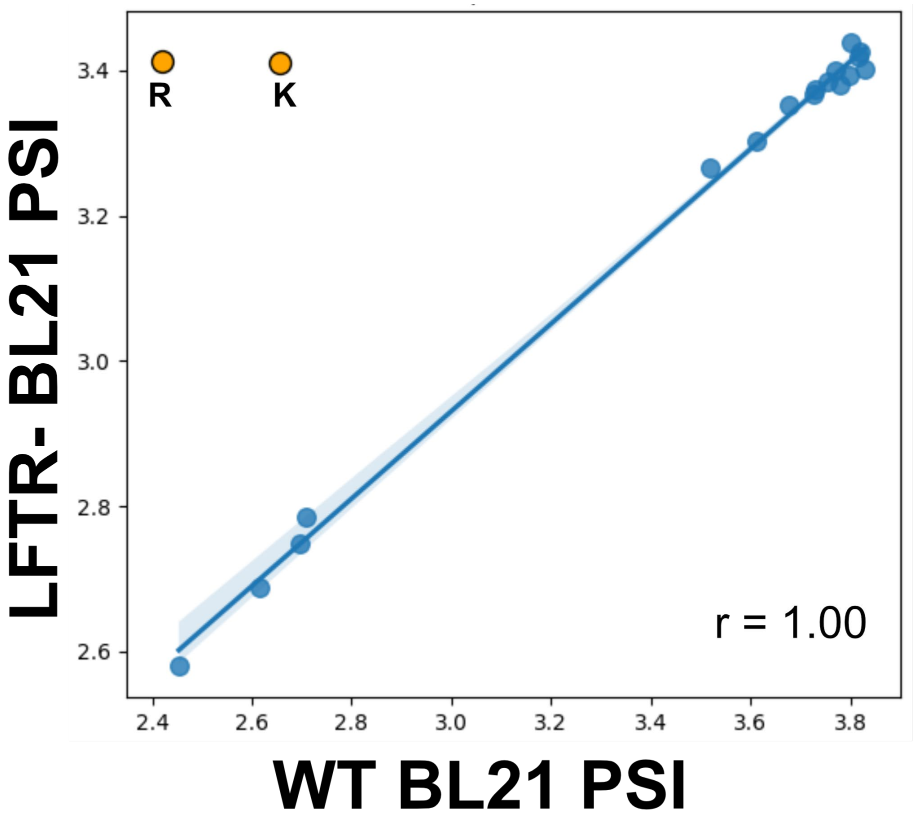

Supplemental Figure 4. P1 amino acid correlation between WT BL21 and LFTR- BL21 sorts
Correlation between PSI scores for N-terminal residues between WT BL21 and LFTR- BL21 (r = 1.00) in the 10 million event sort. Arg/Lys/Pro were excluded from regression analysis. Arg and Lys separately highlighted in orange. Ubiquitin upstream of P1 Pro is not fully cleaved, complicating analysis.

bioRxiv preprint doi: https://doi.org/10.1101/2025.05.22.655665; this version posted May 22, 2025. The copyright holder for this preprint (which was not certified by peer review) is the author/funder, who has granted bioRxiv a license to display the preprint in perpetuity. It is made available under aCC-BY 4.0 International license.

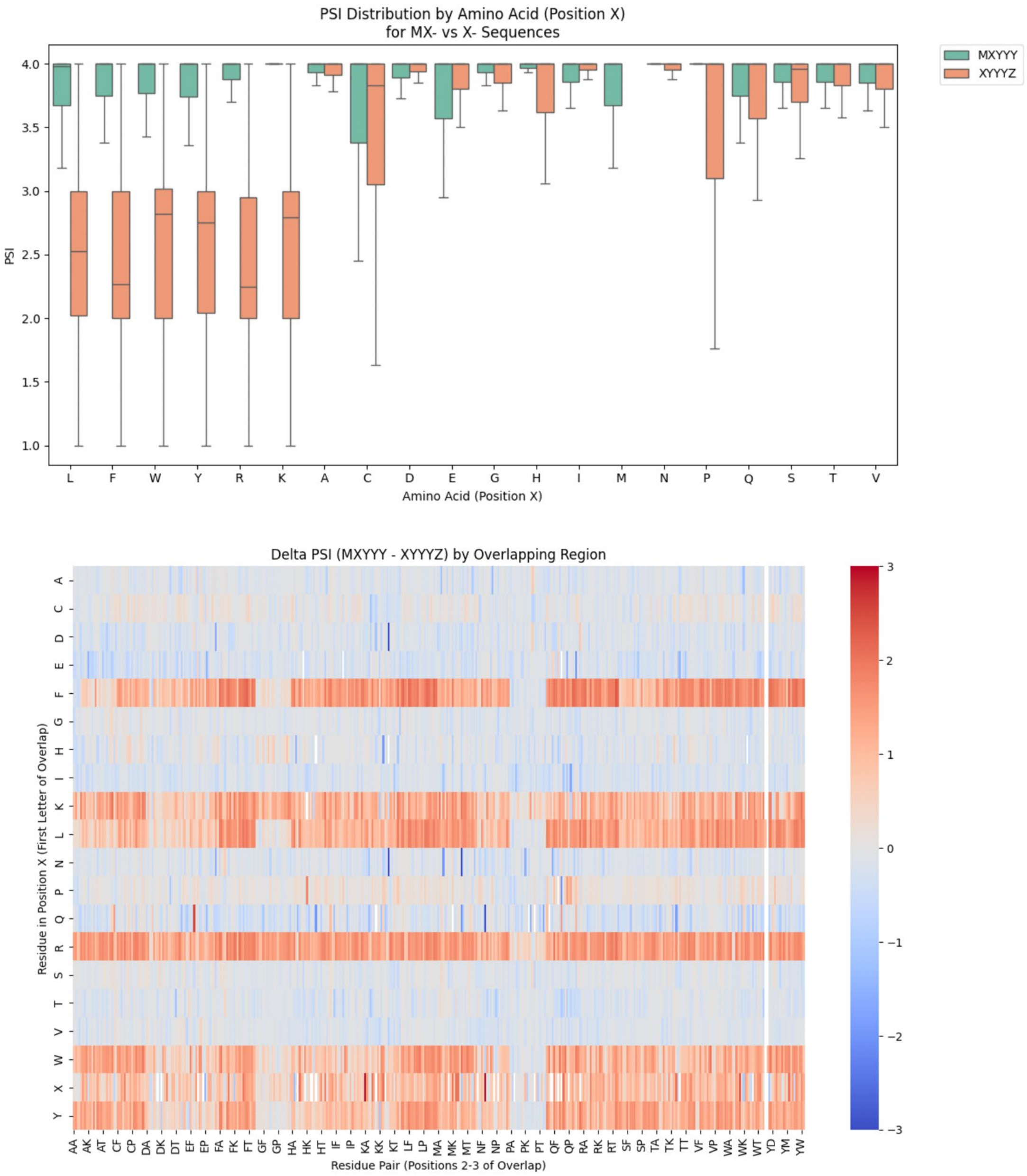

Supplemental Figure 5. MX- Motifs do not show similar PSI distributions for P1 LFWYRK motifs. (Top) Mean PSI boxplots for residues in position X from the 10M event sort. MXYYY is in green and XYYYZ is in orange. (Bottom) Change in PSI between MXYYY and XYYYZ datasets, with the X-position on the y-axis and the first two Y positions in the x-axis.

bioRxiv preprint doi: https://doi.org/10.1101/2025.05.22.655665; this version posted May 22, 2025. The copyright holder for this preprint (which was not certified by peer review) is the author/funder, who has granted bioRxiv a license to display the preprint in perpetuity. It is made available under aCC-BY 4.0 International license.

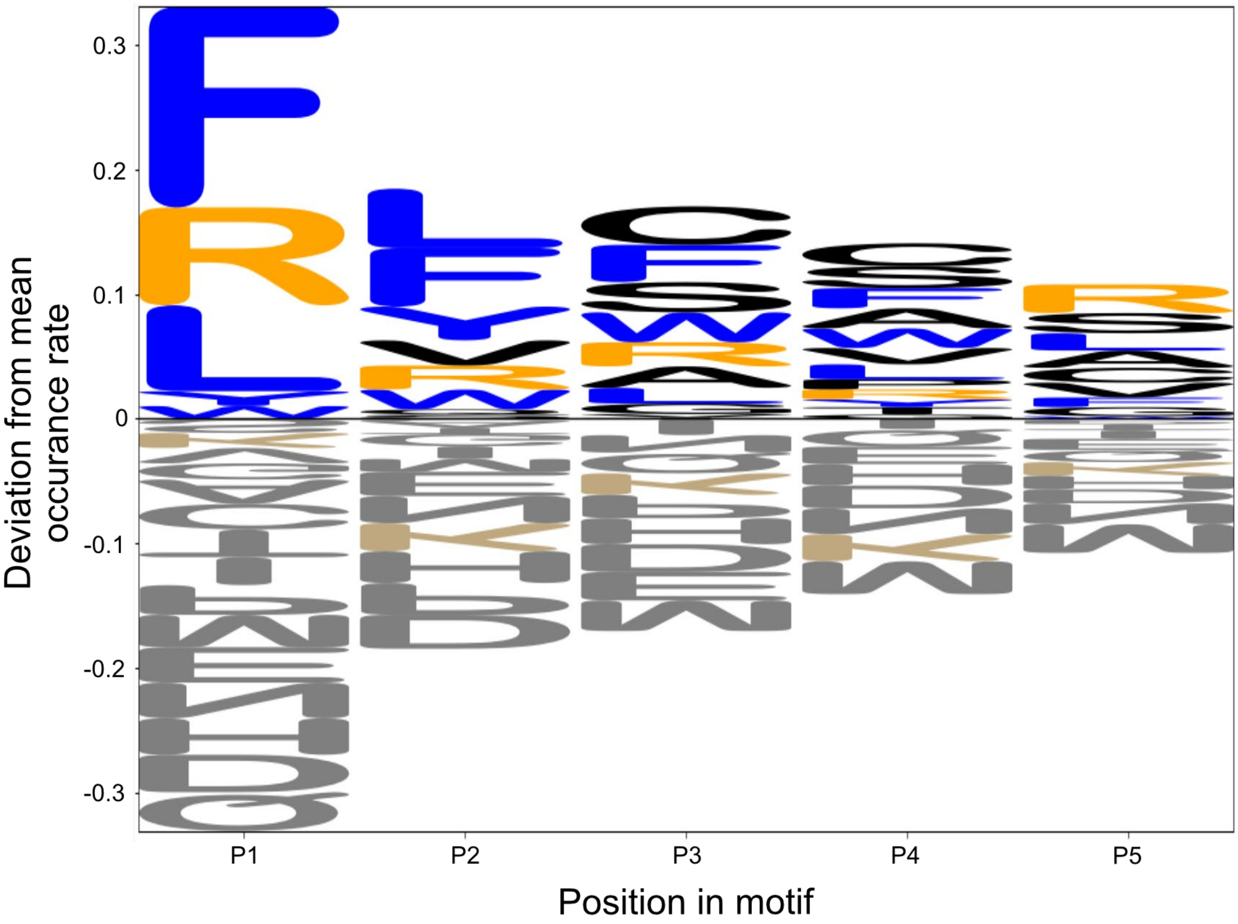

Supplemental Figure 6. Bulky and positively charged amino acids are enriched in low stability sequences. Weblogo depicting the difference in enrichment for each amino acid in each of the five positions in low PSI (PSI <2) samples compared to the entire dataset.

bioRxiv preprint doi: https://doi.org/10.1101/2025.05.22.655665; this version posted May 22, 2025. The copyright holder for this preprint (which was not certified by peer review) is the author/funder, who has granted bioRxiv a license to display the preprint in perpetuity. It is made available under aCC-BY 4.0 International license.

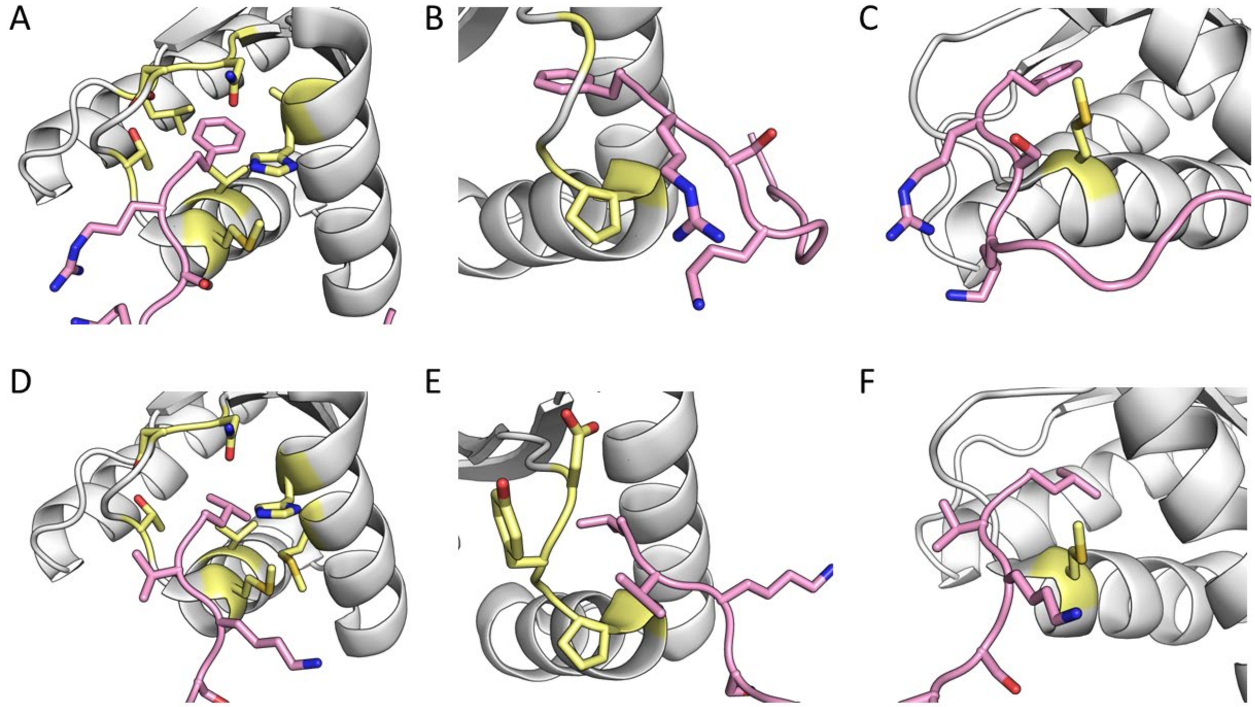

Supplemental Figure 7. Structures of ClpS bound to peptide substrates provide limited insight into the molecular basis of substrate specificity downstream of P1. A-C: Structures of ClpS bound to Fpep with nearby ClpS residues highlighted in yellow for (A) P1, (B) P2, and (C) P3. (PDB ID: 2WA8) D-F: Structures of ClpS bound to Lpep with nearby ClpS residues highlighted in yellow for (D) P1, (E) P2, and (F) P3. (PDB ID: 2W9R). No interacting residues within 4 A were obtained for P4 and P5 of the peptides.

bioRxiv preprint doi: https://doi.org/10.1101/2025.05.22.655665; this version posted May 22, 2025. The copyright holder for this preprint (which was not certified by peer review) is the author/funder, who has granted bioRxiv a license to display the preprint in perpetuity. It is made available under aCC-BY 4.0 International license.

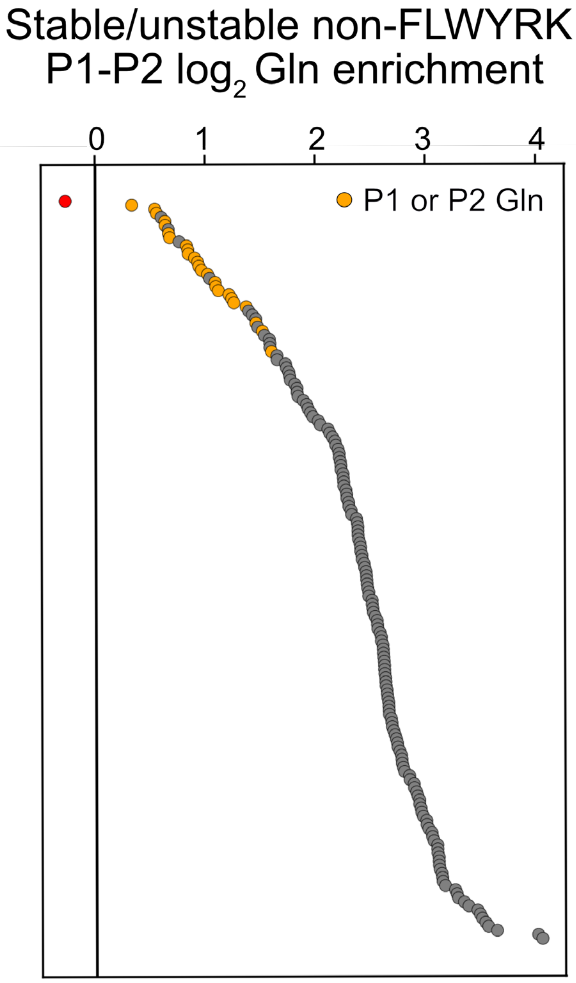

Supplemental Figure 8. P1/P2 Gln is noticeably enriched in low stability P1-P2 motifs that do not have P1 FLWYRK. Log_2 enrichment of P1-P2 motifs in low stability (PSI <2) motifs relative to high stability (PSI >3) motifs where FLWYRK are excluded from P1. Motifs with Gln in P1 or P2 are in orange, QQ is in red.

bioRxiv preprint doi: https://doi.org/10.1101/2025.05.22.655665; this version posted May 22, 2025. The copyright holder for this preprint (which was not certified by peer review) is the author/funder, who has granted bioRxiv a license to display the preprint in perpetuity. It is made available under aCC-BY 4.0 International license.

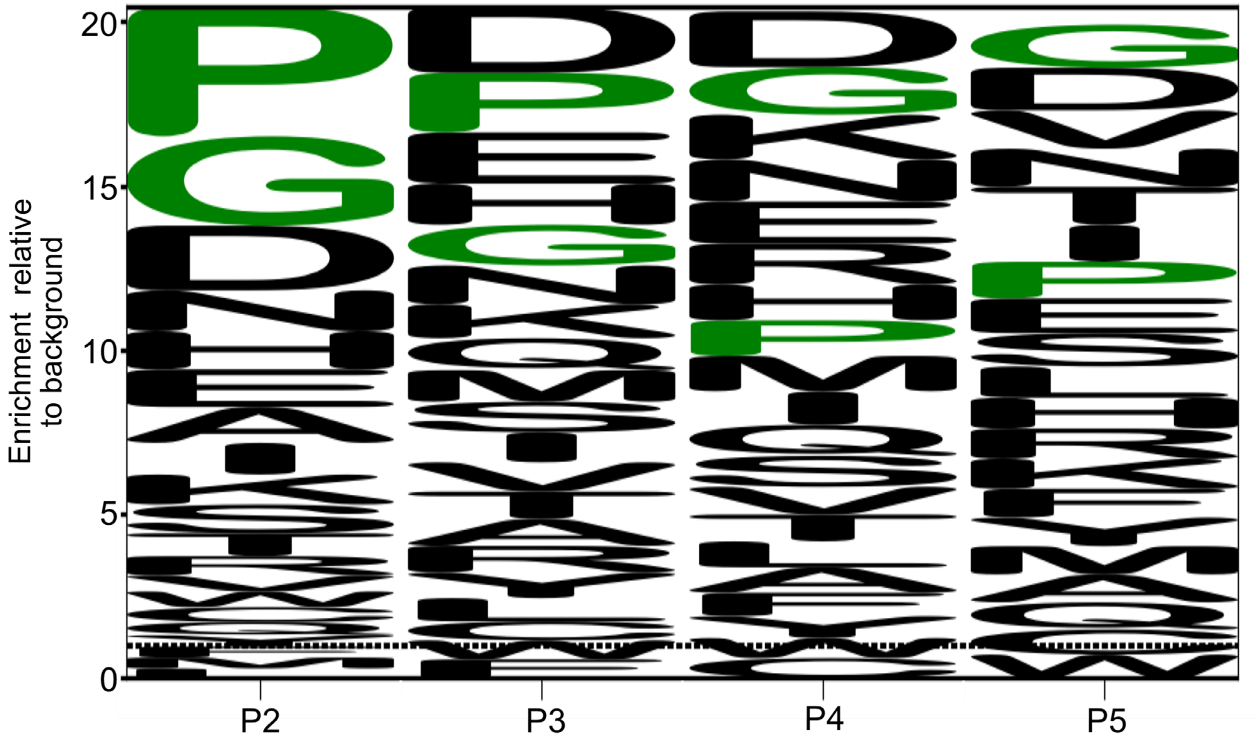

Supplemental Figure 9. Glycine and proline are highly enriched in high stability P1 FLWYRK sequences. A P2-P5 WebLogo showing high stability P1 FLWYRK (PSI >3) sequence enrichment relative to the entire wild-type dataset for each of the five positions studied. Glycine and proline highlighted in green.

bioRxiv preprint doi: https://doi.org/10.1101/2025.05.22.655665; this version posted May 22, 2025. The copyright holder for this preprint (which was not certified by peer review) is the author/funder, who has granted bioRxiv a license to display the preprint in perpetuity. It is made available under aCC-BY 4.0 International license.

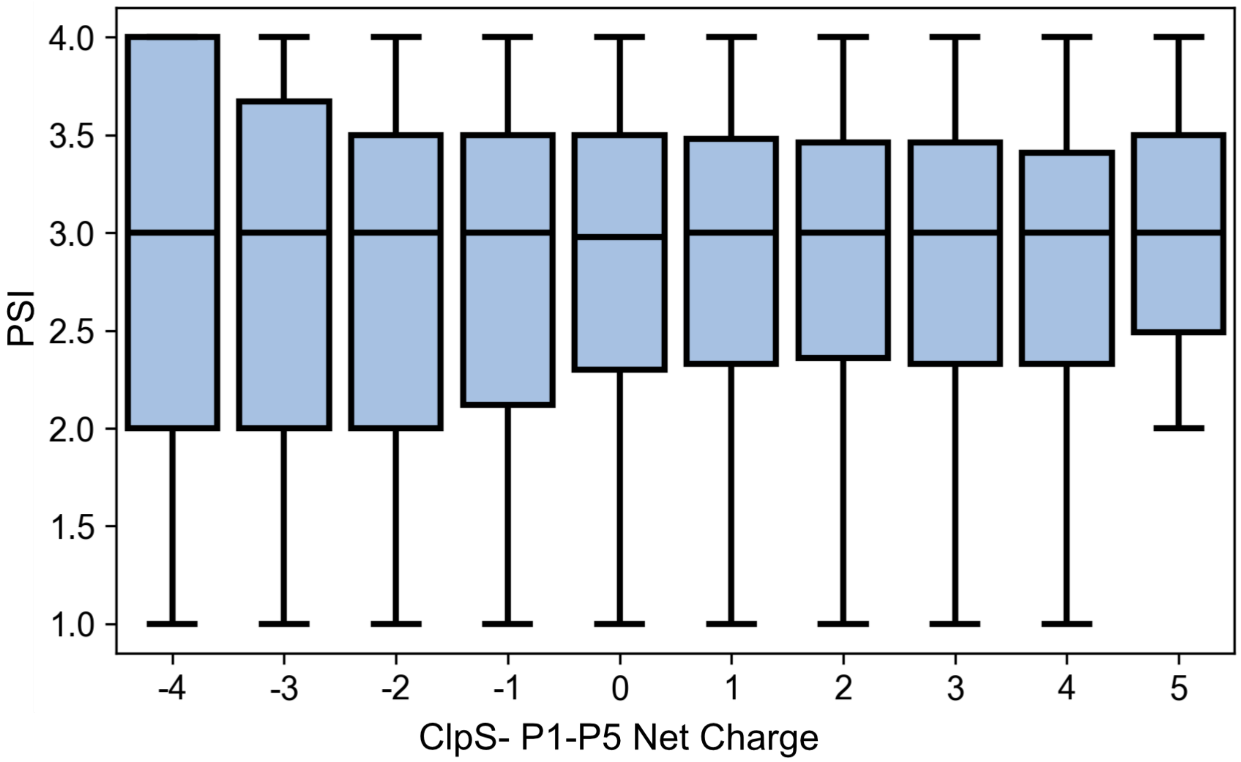

Supplemental Figure 10. ClpS- sequences show charge parity. P1-P5 net charge heatmap for data collected in ClpS- Bl21.

bioRxiv preprint doi: https://doi.org/10.1101/2025.05.22.655665; this version posted May 22, 2025. The copyright holder for this preprint (which was not certified by peer review) is the author/funder, who has granted bioRxiv a license to display the preprint in perpetuity. It is made available under aCC-BY 4.0 International license.

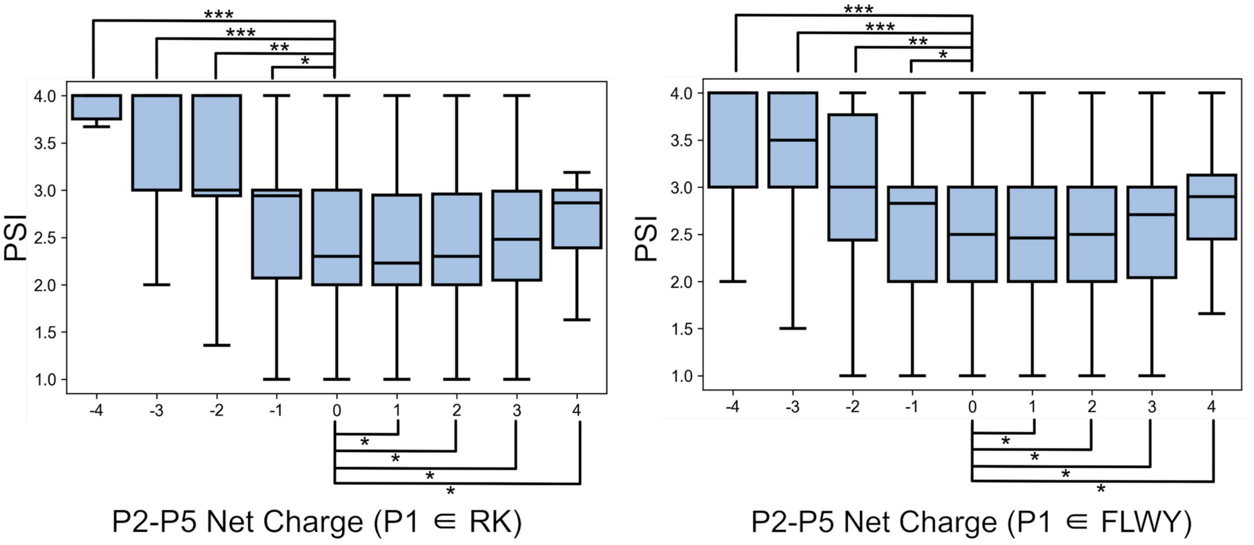

Supplemental Figure 11. Separation of charged and bulky P1 residue net charge heatmaps. All p-values in comparison to 0 P2-P5 Gly/Ser show p <0.05. p-values evaluated using a Mann-Whitney U test, effect size magnitude (ES) ranges from 0 to 1, as evaluated using Cliff's delta. * = p <0.05, ES 0-0.25; ** = p <0.05, ES 0.25-0.50, *** = p <0.05, ES 0.50-0.75, **** = p <0.05, ES 0.75-1.0

bioRxiv preprint doi: https://doi.org/10.1101/2025.05.22.655665; this version posted May 22, 2025. The copyright holder for this preprint (which was not certified by peer review) is the author/funder, who has granted bioRxiv a license to display the preprint in perpetuity. It is made available under aCC-BY 4.0 International license.

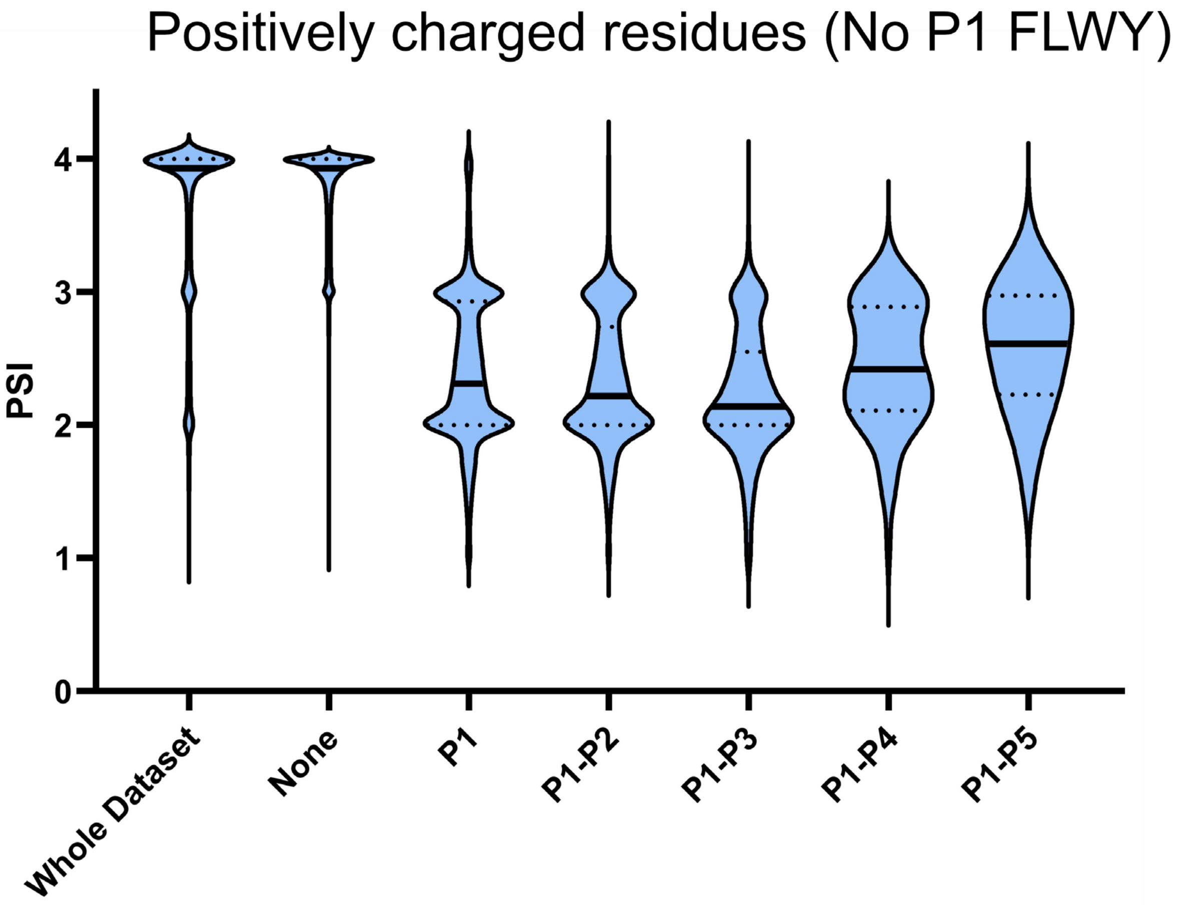

Supplemental Figure 12. Multiple positively charged residues at and near P1 lead to low PSI sequences. Violin plot depicting the PSI distribution in wild-type BL21 for sequences with one or more RK residues in series at and adjacent to the N-terminus.

bioRxiv preprint doi: https://doi.org/10.1101/2025.05.22.655665; this version posted May 22, 2025. The copyright holder for this preprint (which was not certified by peer review) is the author/funder, who has granted bioRxiv a license to display the preprint in perpetuity. It is made available under aCC-BY 4.0 International license.

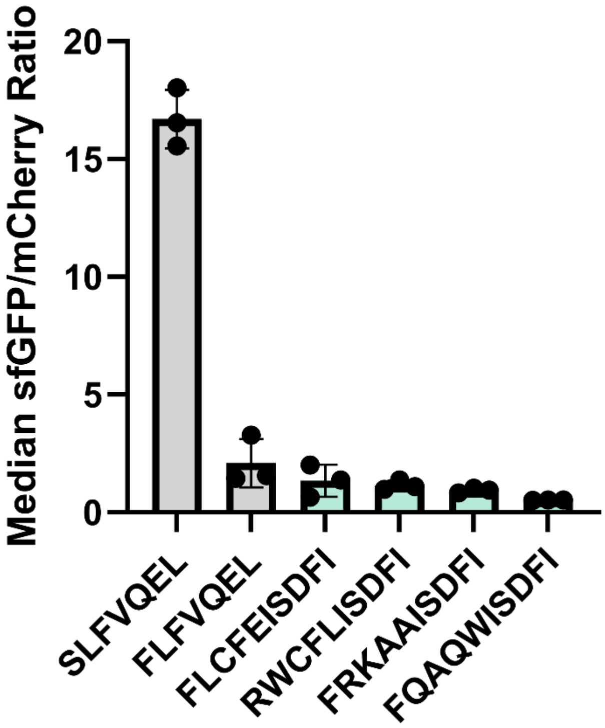

Supplemental Figure 13. Sequences with multiple FLWYRK(Q) residues show low stability. Flow cytometry data from the protein stability assay depicting sequences carrying multiple FLWYRK or P2 Gln residues exhibited low sfGFP:mCherry ratios relative to literature controls.

bioRxiv preprint doi: https://doi.org/10.1101/2025.05.22.655665; this version posted May 22, 2025. The copyright holder for this preprint (which was not certified by peer review) is the author/funder, who has granted bioRxiv a license to display the preprint in perpetuity. It is made available under aCC-BY 4.0 International license.

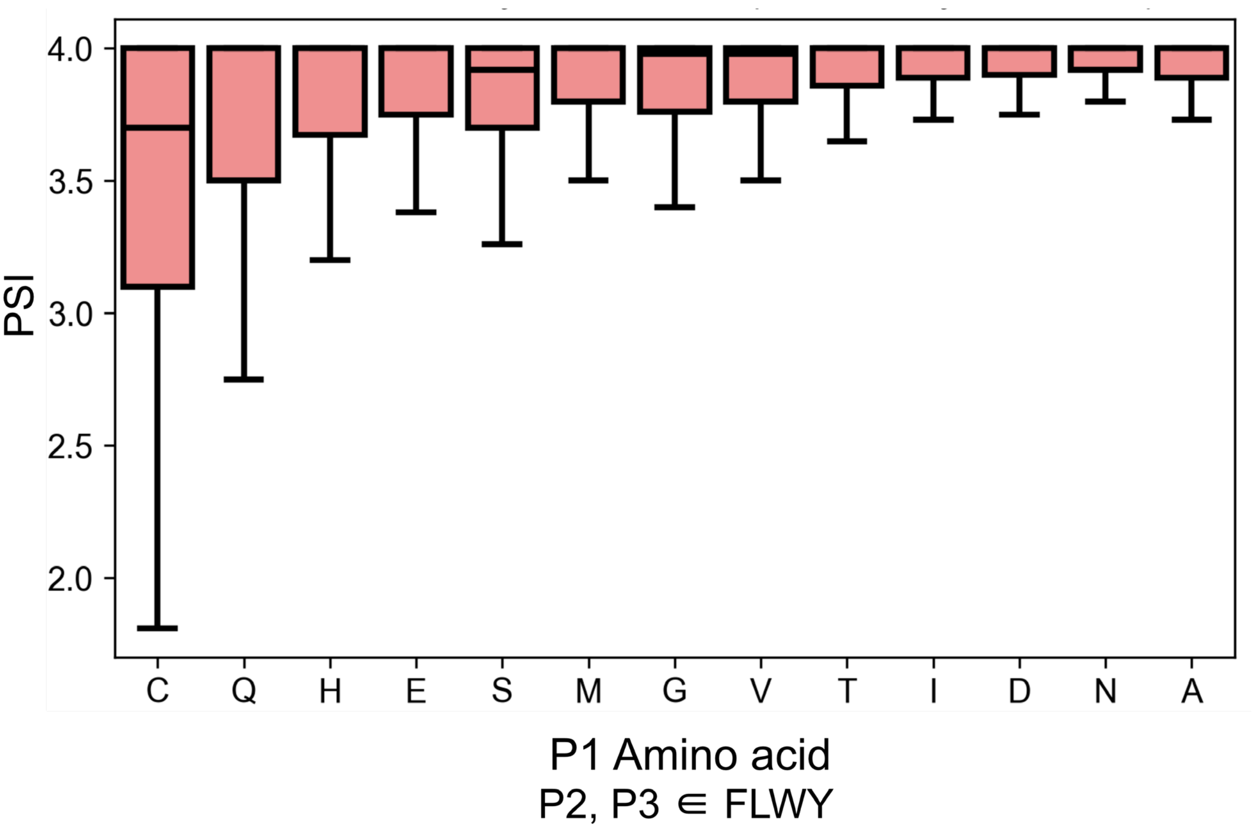

Supplemental Figure 14. P1 Cys is the most destabilizing non FLWYRK residue when P2 and P3 are bulky. Boxplot showing PSI distributions for non-P1 FLWYRKP motifs that have P2 and P3 residues in FLWY.

bioRxiv preprint doi: https://doi.org/10.1101/2025.05.22.655665; this version posted May 22, 2025. The copyright holder for this preprint (which was not certified by peer review) is the author/funder, who has granted bioRxiv a license to display the preprint in perpetuity. It is made available under aCC-BY 4.0 International license.

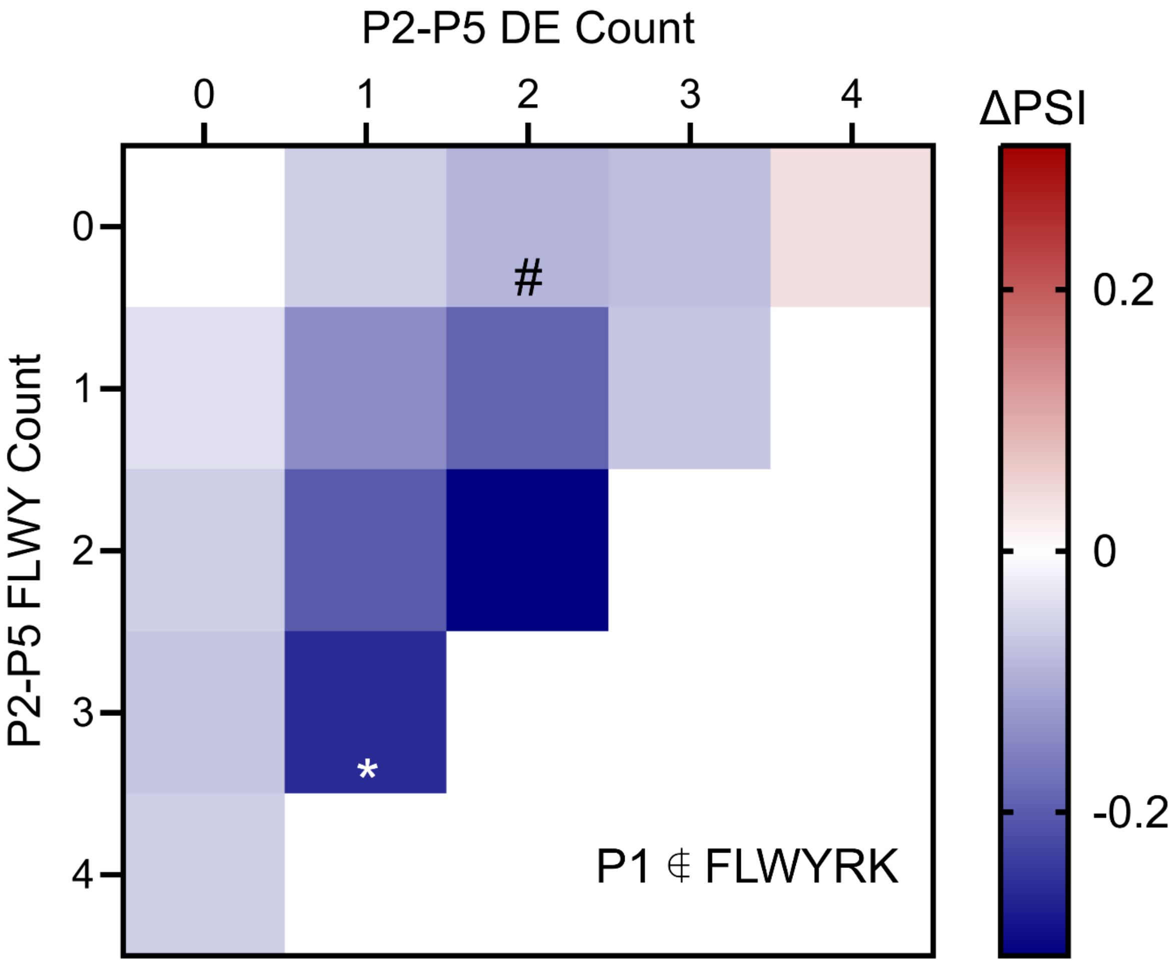

Supplemental Figure 15. For WT sequences with canonically stable P1, the largest PSI drops are observed for motifs with both bulky and negatively charged P2-P5 residues. Change in PSI is relative to the 0 P2-P5 FLWY, 0 P2-P5 DE dataset (mean PSI = 3.78). All p-values relative to (0,0) are p <0.5 except cells marked with # which are p >0.05. * = ES > 0.25.

bioRxiv preprint doi: https://doi.org/10.1101/2025.05.22.655665; this version posted May 22, 2025. The copyright holder for this preprint (which was not certified by peer review) is the author/funder, who has granted bioRxiv a license to display the preprint in perpetuity. It is made available under aCC-BY 4.0 International license.

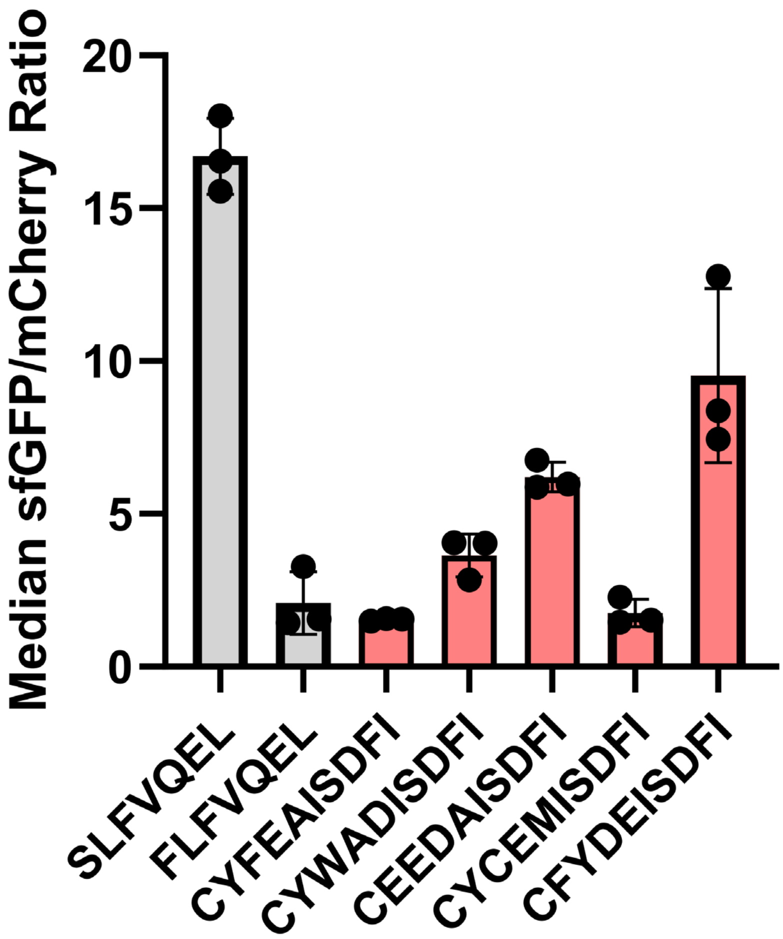

Supplemental Figure 16. Full panel P1 Cys screen reveals putative N-degrons. Flow cytometry data collected from biological triplicates for the full panel of Nt-Cys screened sequences.

bioRxiv preprint doi: https://doi.org/10.1101/2025.05.22.655665; this version posted May 22, 2025. The copyright holder for this preprint (which was not certified by peer review) is the author/funder, who has granted bioRxiv a license to display the preprint in perpetuity. It is made available under aCC-BY 4.0 International license.

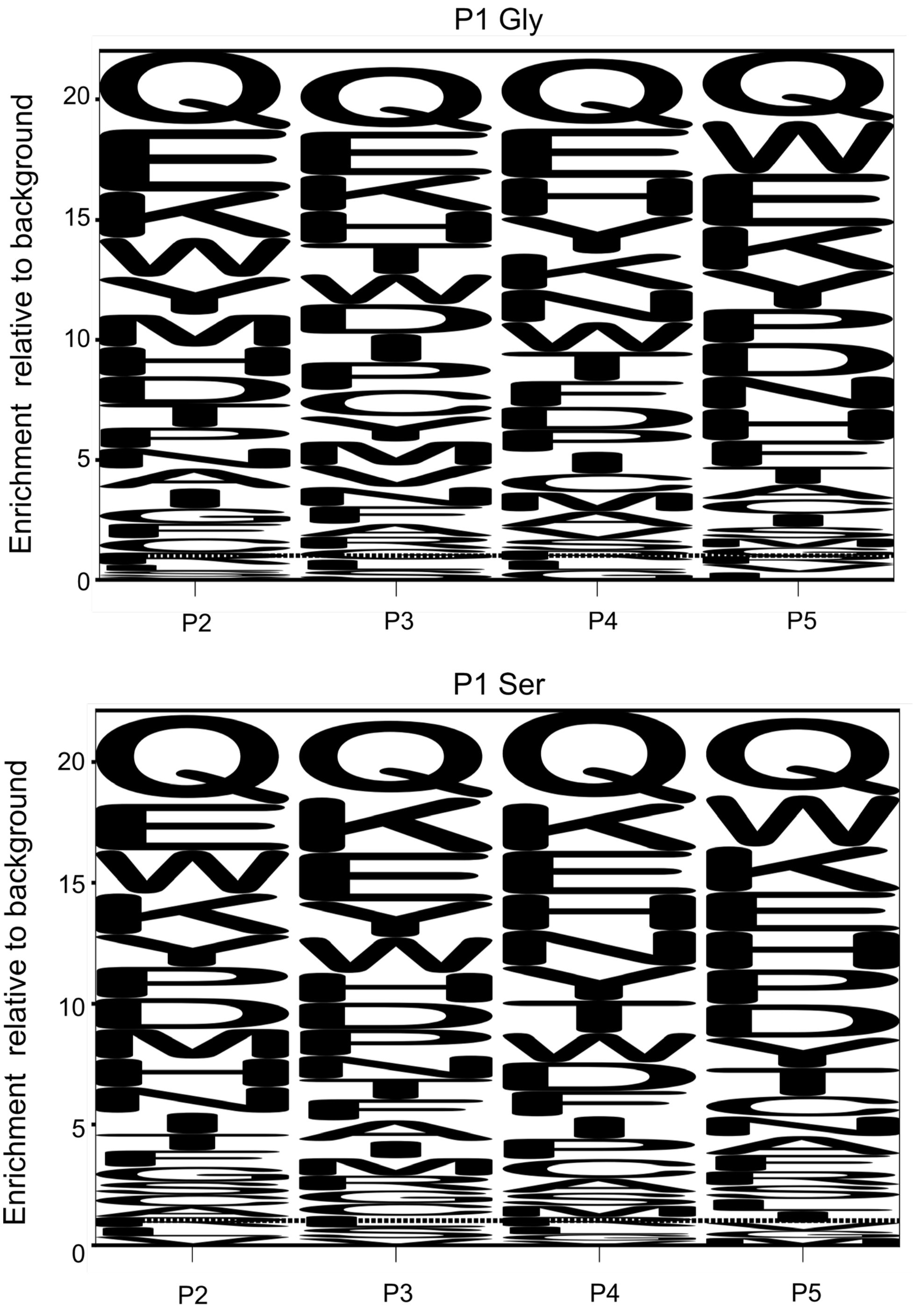

Supplemental Figure 17. Enriched residues in low stability sequences for P1 Gly and P1 Ser. Weblogo depicting the enrichment of amino acids relative to the whole population for the lowest 20,000 PSI sequences with P1 Gly or Ser.

bioRxiv preprint doi: https://doi.org/10.1101/2025.05.22.655665; this version posted May 22, 2025. The copyright holder for this preprint (which was not certified by peer review) is the author/funder, who has granted bioRxiv a license to display the preprint in perpetuity. It is made available under aCC-BY 4.0 International license.

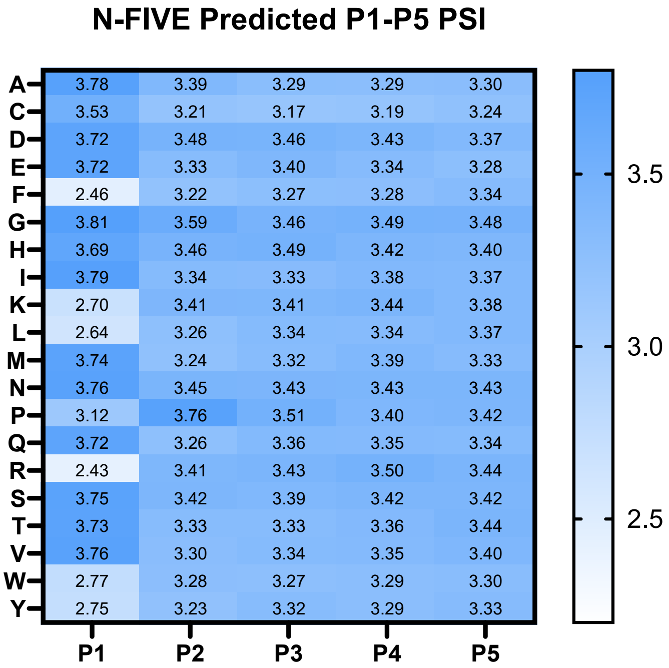

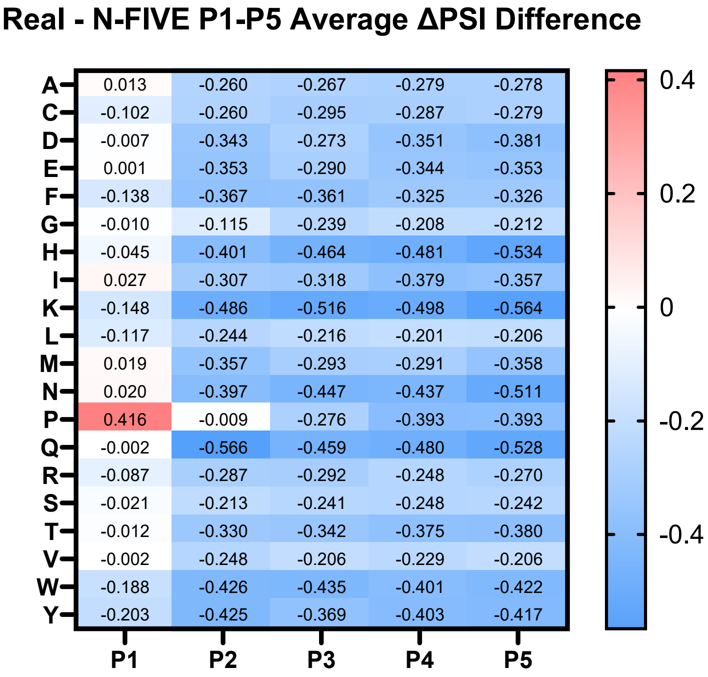

Supplemental Figure 18. N-FIVE PSI predictions. (top) Wild-type BL21 mean PSI heatmap for all 3.2 million sequences as predicted by N-FIVE. (bottom) Change in PSI for each cell between the collected WT BL21 dataset and the N-FIVE predicted dataset.

bioRxiv preprint doi: https://doi.org/10.1101/2025.05.22.655665; this version posted May 22, 2025. The copyright holder for this preprint (which was not certified by peer review) is the author/funder, who has granted bioRxiv a license to display the preprint in perpetuity. It is made available under aCC-BY 4.0 International license.

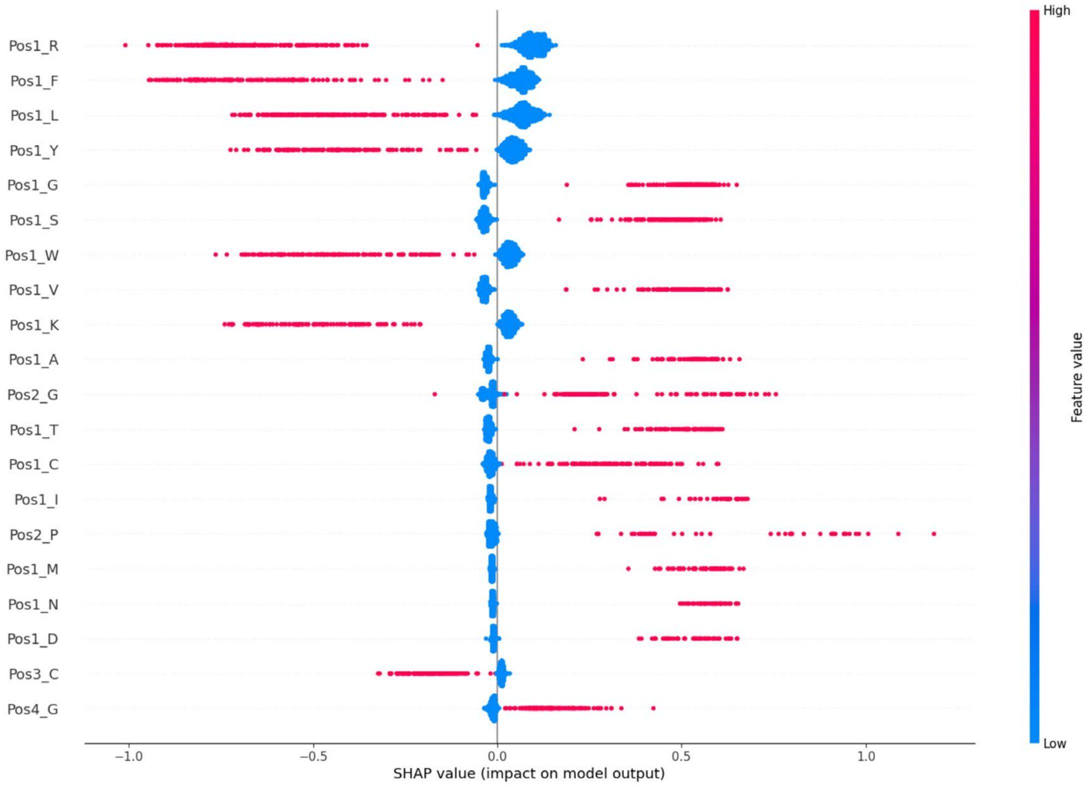

Supplemental Figure 19. Individual matrix components for N-FIVE predictions. SHAP values showing the contribution of the 20 most impactful matrix features onto the N-FIVE model.

bioRxiv preprint doi: https://doi.org/10.1101/2025.05.22.655665; this version posted May 22, 2025. The copyright holder for this preprint (which was not certified by peer review) is the author/funder, who has granted bioRxiv a license to display the preprint in perpetuity. It is made available under aCC-BY 4.0 International license.

Supplemental Tables

Supplemental Table 1. Strains

Strain	Description	Key mutations
E. coli BL21 DE3	Standard *E. coli* BL21 D3	N/A
E. coli BL21 DE3 *clpS-*	BL21 with inserted in-frame stop codons for *clpS*	V46*, L47*, Q48*
E. coli BL21 DE3 *aat-*	BL21 with inserted in-frame stop codons for *aat* (LFTR)	L20*, R21*, E22*

Supplemental Table 2. Plasmids

pEVOL constructs are arabinose inducible, chloramphenicol resistant and pZE constructs are aTc inducible, kanamycin resistant unless listed otherwise.

Plasmid ID	Description	Description
pSS052	pEVOL mCherry-Ub-SLFVQEL-sfGFP-His6x	Dual reporter construct
pSS054	pEVOL mCherry-Ub-FLFVQEL-sfGFP-His6x	Dual reporter construct
pSS060	pEVOL mCherry-ArgRAP2.2_nt10-sfGFP-His6x	Dual reporter construct
pSS129	pEVOL mCherry-DPSnt6-16-sfGFP-His6x	Dual reporter construct
pSS142	pEVOL mCherry-Asp-Bgalnt10-sfGFP-His6x	Dual reporter construct
pSS168	pZE Ubp1 w TetR	scUlp1 protease construct
pSS303	pEVOL mCherry-Ub-(P1-P5NNK)-RAP2.2nt10-sfGFP	Dual reporter library with mutagenized P1-P5 residues
pSS0489	pEVOL mCherry-Ub-CFYEAISDFI-sfGFP	Dual reporter construct
pSS0490	pEVOL mCherry-Ub-CYWADISDFI-sfGFP	Dual reporter construct
pSS0491	pEVOL mCherry-Ub-CEEDAISDFI-sfGFP	Dual reporter construct
pSS0492	pEVOL mCherry-Ub-CYCEMISDFI-sfGFP	Dual reporter construct
pSS0493	pEVOL mCherry-Ub-CFYDEISDFI-sfGFP	Dual reporter construct
pSS0494	pEVOL mCherry-Ub-PYFDEISDFI-sfGFP	Dual reporter construct
pSS0495	pEVOL mCherry-Ub-IFFAEISDFI-sfGFP	Dual reporter construct
pSS0496	pEVOL mCherry-Ub-IWYKHISDFI-sfGFP	Dual reporter construct
pSS0497	pEVOL mCherry-Ub-FQKQKISDFI-sfGFP	Dual reporter construct
pSS0498	pEVOL mCherry-Ub-RQWYHISDFI-sfGFP	Dual reporter construct
pSS0499	pEVOL mCherry-Ub-FDEDEISDFI-sfGFP	Dual reporter construct
pSS0500	pEVOL mCherry-Ub-FDEAAISDFI-sfGFP	Dual reporter construct
pSS0501	pEVOL mCherry-Ub-FAAAAISDFI-sfGFP	Dual reporter construct

bioRxiv preprint doi: https://doi.org/10.1101/2025.05.22.655665; this version posted May 22, 2025. The copyright holder for this preprint (which was not certified by peer review) is the author/funder, who has granted bioRxiv a license to display the preprint in perpetuity. It is made available under aCC-BY 4.0 International license.

pSS0502	pEVOL mCherry-Ub-FRKAAISDFI-sfGFP	Dual reporter construct
pSS0504	pEVOL mCherry-Ub-GLVRGISDFI-sfGFP	Dual reporter construct
pSS0505	pEVOL mCherry-Ub-GLRLRISDFI-sfGFP	Dual reporter construct
pSS0506	pEVOL mCherry-Ub-LVRLGISDFI-sfGFP	Dual reporter construct
pSS0507	pEVOL mCherry-Ub-GSVLRISDFI-sfGFP	Dual reporter construct
pSS0508	pEVOL mCherry-Ub-HWMQKISDFI-sfGFP	Dual reporter construct
pSS0509	pEVOL mCherry-Ub-NIQMKISDFI-sfGFP	Dual reporter construct
pSS0510	pEVOL mCherry-Ub-SLWKYISDFI-sfGFP	Dual reporter construct
pSS0511	pEVOL mCherry-Ub-SYMMDISDFI-sfGFP	Dual reporter construct
pSS0512	pEVOL mCherry-Ub-IKKTQISDFI-sfGFP	Dual reporter construct
pSS0513	pEVOL mCherry-Ub-LAASYISDFI-sfGFP	Dual reporter construct
pSS0514	pEVOL mCherry-Ub-LDRNSISDFI-sfGFP	Dual reporter construct
pSS0515	pEVOL mCherry-Ub-LAVPRISDFI-sfGFP	Dual reporter construct
pSS0516	pEVOL mCherry-Ub-LGPRSISDFI-sfGFP	Dual reporter construct
pSS0517	pEVOL mCherry-Ub-LGDSVISDFI-sfGFP	Dual reporter construct
pSS0518	pEVOL mCherry-Ub-QQCKWISDFI-sfGFP	Dual reporter construct
pSS0519	pEVOL mCherry-Ub-HQAQWISDFI-sfGFP	Dual reporter construct
pSS0520	pEVOL mCherry-Ub-FQAQWISDFI-sfGFP	Dual reporter construct
pSS0521	pEVOL mCherry-Ub-WGAAVISDFI-sfGFP	Dual reporter construct
pSS0522	pEVOL mCherry-Ub-RGAAVISDFI-sfGFP	Dual reporter construct
pSS0523	pEVOL mCherry-Ub-LGAAVISDFI-sfGFP	Dual reporter construct
pSS0524	pEVOL mCherry-Ub-FLCFEISDFI-sfGFP	Dual reporter construct
pSS0525	pEVOL mCherry-Ub-RWCFLISDFI-sfGFP	Dual reporter construct

bioRxiv preprint doi: https://doi.org/10.1101/2025.05.22.655665; this version posted May 22, 2025. The copyright holder for this preprint (which was not certified by peer review) is the author/funder, who has granted bioRxiv a license to display the preprint in perpetuity. It is made available under aCC-BY 4.0 International license.

Supplemental Table 3. Important gene sequences

Genetic construct	DNA Sequence
Dual fluorescent reporter (mCherry-Ub-RCKGLAALPHS-sfGFP-His6x	ATGGTGAGCAAGGGCGAGGaggataacatggccatcatcaaggagt<br>tcatgcgcttcaaggtgcacatggagggctccgtgaacggccacgagttcgagat<br>cgagggcgagggcgagggccgcccctacgagggcacccagaccgccaagctg<br>aaggtgaccaagggtggccccctgcccttcgcctgggacatcctgtcccctcag<br>ttcatgtacggctccaaggcctacgtgaagcaccccgccgacatccccgacta<br>cttgaagctgtccttccccgagggcttcaagtgggagcgcgtgatgaacttcgagg<br>acggcggcgtggtgaccgtgacccaggactcctcactgcaggacggcgagttca<br>tctacaaggtgaagctgcgcggcaccaacttcccctccgacggccccgtaatg<br>cagaagaagaccatgggctgggaggcctcctccgagcggatgtaccccgagga<br>cggcgccctgaagggcgagatcaagcagaggctgaagctgaaggacggcggc<br>cactacgacgctgaggtcaagaccacctacaaggccaagaagcccgtgcagc<br>tgcccggcgcctacaacgtcaacatcaagttggacatcacctcccacaacgag<br>gactacaccatcgtggaacagtacgaacgcgctgagggccgccactccaccg<br>gcggcATGGACGAGCTGTACAAGCAGATTTTTGTGAAGACTT<br>TAACAGGTAAGACGATTACCCTGGAGGTGGAGTCCTCGG<br>ACACCATCGATAATGTAAAATCAAAAATCCAAGATAAGGAA<br>GGAATCCCTCCAGACCAGCAACGTCTGATTTTCGCAGGTA<br>AACAACTGGAGGATGGTCGCACGCTTTCGGACTACAACAT<br>CCAGAAAGAATCTACCCTTCATTTGGTTCTGCGTCTGCGTG<br>GAGGACGCTGCAAAGGACTTGCAGCACTGCCCCATTCCg<br>catccaagggcgaggagctctttactggcgtagtaccaattctcgtagagctcgat<br>ggcgatgtaaatggccataagttttccgtacgcggcgagggcgagggcgatgcaa<br>ctaacggcaagctcactctcaagtttatttgtactactggcaagctcccagtacc<br>atggccaactctcgtaactactctgacctatggcgtacaatgtttttcccgctatc<br>cagatcacatgaagcaacatgatttttttaagtccgcaatgccagagggctatgta<br>caagagcgcactattagctttaaggatgatggcacctataagactcgcgcagag<br>gtaaagtttgagggcgatactctcgtaaatcgcattgagctcaagggcattgatttt<br>aaggaggatggcaatattctcggccataagctggagtataatttcaattcccataa<br>tgtatacattaccgcagataagcaaaagaatggcattaaggcgaattttaagattc<br>gccataatgtggaggatggctccgtacaactcgcagatcattatcaacaaaata<br>ctccaattggcgatggcccagtactcctcccagataatcattatctctccactca<br>atccgtgctctccaaagatccaaatgagaagcgcgatcacatggtactcctgga<br>gtttgtaactgcagcaggcattactcatggcatggatgagctctataagctcgagc<br>accaccaccaccaccactaa

bioRxiv preprint doi: https://doi.org/10.1101/2025.05.22.655665; this version posted May 22, 2025. The copyright holder for this preprint (which was not certified by peer review) is the author/funder, who has granted bioRxiv a license to display the preprint in perpetuity. It is made available under aCC-BY 4.0 International license.

scUbp1, truncated	ATGGGGAGTGGGTCTTTCATTGCTGGGCTTGTCAACGATG GTAATACGTGTTTTATGAACTCGGTTCTTCAGTCCCTTGCTA GTAGCCGTGAACTTATGGAGTTTTTGGATAATAATGTAATCC GTACATATGAAGAAATTGAACAGAACGAGCACAATGAGGA AGGTAATGGCCAAGAGAGCGCACAAGATGAGGCAACTCA CAAAAAAAACACTCGCAAGGGAGGTAAGGTCTATGGGAA GCATAAAAAGAAATTAAACCGCAAATCTTCTAGCAAGGAA GACGAAGAAAAGTCGCAAGAACCAGACATTACGTTTTCGG TGGCGTTGCGTGATCTGCTGAGCGCATTAAATGCTAAGTAT TATCGCGACAAACCCTACTTTAAGACTAACTCTTTATTAAAA GCGATGAGCAAGTCCCCGCGCAAAAATATCTTGCTTGGGT ACGATCAAGAAGACGCTCAGGAATTTTTTCAAAACATTCTT GCGGAGTTAGAATCTAATGTCAAGTCGTTAAACACAGAAA AGCTTGATACTACACCGGTAGCCAAGTCCGAACTTCCAGA CGATGCTCTGGTTGGCCAATTAAACCTTGGTGAGGTAGGC ACCGTGTACATTCCCACAGAACAAATTGACCCCAATTCGA TTTTACATGACAAATCGATTCAAAACTTTACCCCCTTTAAAC TGATGACCCCGTTGGATGGGATCACGGCTGAGCGCATCG GCTGCCTGCAATGCGGAGAGAACGGGGGAATTCGCTACA GTGTTTTCAGCGGATTAAGTTTGAACCTGCCGAATGAAAAT ATTGGAAGCACTCTTAAACTGTCCCAGTTACTGTCCGATTG GTCGAAACCCGAGATTATCGAGGGTGTTGAATGCAACCGT TGCGCTTTAACAGCTGCGCACTCACACTTGTTTGGCCAATT AAAGGAGTTTGAGAAGAAACCTGAAGGCTCGATTCCCGAA AAACTTATTAATGCCGTAAAGGACCGCGTGCACCAGATCG AAGAGGTCTTGGCAAAGCCGGTTATCGACGATGAAGATTA TAAAAAATTGCATACTGCGAATATGGTCCGCAAGTGTTCAA AAAGTAAACAAATTCTTATCTCTCGTCCACCACCTTTGTTGT CTATTCATATCAACCGCTCTGTTTTCGACCCGCGCACCTA CATGATTCGCAAGAACAACTCCAAGGTTTTGTTCAAGTCAC GCTTGAACCTGGCACCCTGGTGCTGTGATATCAACGAAAT CAATCTTGACGCACGCCTTCCGATGTCGAAGAAGGAAAAA GCAGCTCAACAAGATTCTTCTGAAGACGAGAACATTGGCG GAGAGTACTATACTAAATTGCATGAACGTTTTGAGCAGGAG TTTGAAGATTCTGAAGAAGAGAAGGAATACGATGATGCAG AGGGTAATTATGCATCGCATTATAACCATACCAAGGACATC TCCAACTACGATCCATTGAATGGAGAAGTCGACGGTGTGA CTTCCGATGATGAGGATGAATACATTGAAGAGACAGACGC GTTGGGGAATACCATCAAAAAACGTATTATTGAACACTCCG ACGTGGAGAACGAAAACGTGAAGGATAATGAAGAACTTCA GGAGATCGATAACGTTAGCTTGGATGAGCCAAAAATTAAT GTCGAGGACCAGCTTGAAACGAGTTCTGATGAGGAAGAC GTTATTCCTGCTCCACCCATCAACTACGCTCGCAGCTTTA GTACGGTCCCAGCGACCCCTTTAACTTACTCTTTGCGCAG

bioRxiv preprint doi: https://doi.org/10.1101/2025.05.22.655665; this version posted May 22, 2025. The copyright holder for this preprint (which was not certified by peer review) is the author/funder, who has granted bioRxiv a license to display the preprint in perpetuity. It is made available under aCC-BY 4.0 International license.

	CGTCATCGTGCACTATGGGACTCACAACTACGGACATTAT<br>ATTGCATTTCGCAAGTATCGTGGATGTTGGTGGCGCATCTC<br>CGATGAGACGGTCTATGTGGTAGATGAGGCCGAAGTACTG<br>TCAACACCGGGGGTATTTATGCTTTTCTACGAGTATGATTT<br>CGACGAGGAGACCGGAAAAATGAAAGACGACTTAGAAGC<br>TATCCAGAGCAATAATGAGGAAGATGACGAGAAAGAACAG<br>GAACAGAAGGGTGTCCAGGAGCCAAAAGAATCCCAGGAG<br>CAAGGCGAAGGCGAAGAACAAGAAGAAGGGCAAGAGCA<br>AATGAAATTTGAGCGTACGGAGGATCATCGCGACATTTCA<br>GGGAAGGATGTGAATTAA
